# A Significantly High Abundance of “*Candidatus* Liberibacter asiaticus” in Citrus Fruit Pith: *in planta* Transcriptome and Anatomical Analyses

**DOI:** 10.3389/fmicb.2021.681251

**Published:** 2021-06-11

**Authors:** Fang Fang, Hengyu Guo, Anmin Zhao, Tao Li, Huihong Liao, Xiaoling Deng, Meirong Xu, Zheng Zheng

**Affiliations:** ^1^Guangdong Province Key Laboratory of Microbial Signals and Disease Control, South China Agricultural University, Guangzhou, China; ^2^Horticulture Research Institute, Guangxi Academy of Agricultural Sciences, Nanning, China

**Keywords:** citrus huanglongbing, *Candidatus* Liberibacter asiaticus, high abundance, fruit pith, *in planta* transcriptome, anatomical analyses

## Abstract

Huanglongbing, a highly destructive disease of citrus, is associated with the non-culturable phloem-limited α-proteobacterium “*Candidatus* Liberibacter asiaticus” (CLas). The distribution patterns of CLas in infected plant are variable and not consistent, which make the CLas detection and characterization more challenging. Here, we performed a systemic analysis of CLas distribution in citrus branches and fruits of 14 cultivars. A significantly high concentration of CLas was detected in fruit pith (dorsal vascular bundle) of 14 citrus cultivars collected at fruit maturity season. A 2-year monitoring assay of CLas population in citrus branches of “Shatangju” mandarin (*Citrus reticulata* Blanco “Shatangju”) revealed that CLas population already exhibited a high level even before the appearance of visual symptoms in the fruit rind. Quantitative analyses of CLas in serial 1.5-cm segments of fruit piths showed the CLas was unevenly distributed within fruit pith and tended to colonize in the middle or distal (stylar end) regions of pith. The use of CLas-abundant fruit pith for dual RNA-seq generated higher-resolution CLas transcriptome data compared with the leaf samples. CLas genes involved in transport system, flagellar assembly, lipopolysaccharide biosynthesis, virulence, stress response, and cell surface structure, as well as host genes involved in biosynthesis of antimicrobial-associated secondary metabolites, was up-regulated in leaf midribs compared with fruit pith. In addition, CLas infection caused the severe collapse in phloem and callose deposition in the plasmodesmata of fruit pith. The ability of fruit pith to support multiplication of CLas to high levels makes it an ideal host tissue for morphological studies and *in planta* transcriptome analyses of CLas–host interactions.

## Introduction

Huanglongbing (HLB, also called yellow shoot disease) is a highly destructive disease currently threatening citrus production worldwide. It is associated with three unculturable phloem-limited α-proteobacterium, *Candidatus* Liberibacter asiaticus (CLas), “*Ca*. L. africanus,” and “*Ca*. L. americanus” (Jagoueix et al., 1994; Teixeira et al., 2005). The most severe form of the disease is associated with CLas, which is most commonly transmitted by Asian citrus psyllid (*Diaphorina citri* Kuwayama). In China, HLB was first observed in Chaoshan region of Guangdong province over a century ago (Lin, 1956). Literature records indicated the HLB was initially endemic in the coastal region of southern China and spread to other citrus-growing province after the 1970s (Lin, 1956; Zheng et al., 2018). The association of CLas to HLB in China was established in 1996 (Deng and Tang, 1996; Tian et al., 1996). Because of the current inability to culture CLas *in vitro*, the identification of CLas was usually performed with total plant DNA by polymerase chain reaction (PCR) or real-time PCR targeting the specific and conserved genomic loci of CLas genome, mostly in the 16S rRNA gene (Li et al., 2006; Bao et al., 2020) or recently identified ribonucleotide reductase genes (Zheng et al., 2016).

The destructive impact of HLB on cultivation of citrus stems from the impact of the disease on the lifespan of trees, yields, and fruit quality (Bové, 2006; Zheng et al., 2018). Most commercial cultivars are susceptible to severe impacts of the disease (Bové, 2006). Although citrus trees can be affected by HLB at any age, symptoms caused by disease are variable, with differences related to host cultivar and age, time of infection, and season and types of tissues in which symptoms are expressed (Lin, 1956; Zheng et al., 2018). The most characteristic symptoms of HLB included the yellowing of leaves, initially on one or branches among many, asymmetrical blotchy mottling of leaves, unseasonal flowering, small asymmetrical fruit with aborted seeds, uneven initial coloring of fruit, and premature fruit drop (Lin, 1956; Zheng et al., 2018). As the extent of infection increases, leaves on new growth may be atypically small and exhibit symptoms of zinc or manganese deficiency symptoms. Diseased fruits that color unevenly are referred to as “red-nose” fruit, with color changing from green to yellow occurring initially from the calyx (stem) end instead of stylar end, with the stylar end, in most situations, remaining green as fruit mature. Occurrence of “red-nose” fruit was reported as a characteristic HLB symptom observed in “Shatangju” mandarin (*Citrus reticulata* Blanco “Shatangju”) (Zhang et al., 2011).

Although HLB is a systemic disease, CLas is generally unevenly distributed within infected plants (Tatineni et al., 2008; Li et al., 2009; Hartung et al., 2010; Kunta et al., 2014; Ding et al., 2015; Louzada et al., 2016; Fu et al., 2019). It is more frequently found in symptomatic leaves than the asymptomatic leaves from infected trees (Ding et al., 2015; Louzada et al., 2016). Even within the individual symptomatic leaves, the CLas tends to colonize the underside of petioles in preference to the upside of petiole (Ding et al., 2015). An *in planta* distribution analysis of CLas in different tissues from diseased trees has shown that CLas cells were most abundant in the fruit peduncles than in other tissues but absent in the endosperm and embryos of infected seeds (Tatineni et al., 2008). Highest CLas titers have been recorded in fruit peduncles of CLas-infected grapefruit and sweet orange in contrast to low titers in young shoots (Kunta et al., 2014). However, another investigation based on different tissues of six citrus species/hybrids revealed that the CLas population levels in the fruit tissues were significantly less (∼1,000-fold) than those in the midribs, leaf blades, barks, and roots (Li et al., 2009). The pattern of CLas distribution within infected citrus trees could be different according to citrus species/hybrids and disease severity (Li et al., 2009).

Currently, the CLas has not been cultured *in vitro* yet. The characterization of CLas was mainly relied on the analyses of CLas-infected host tissue. However, the uneven distribution of CLas and the highly variable bacterial titers in infected plant makes the CLas research more challenging. A reliable CLas characterization requires that host tissue contains a stable high CLas concentration. In this study, the distribution of CLas was thoroughly analyzed in citrus branches and fruits of 14 commercial cultivars collected in southern China. Significantly high levels of CLas population were detected in fruit pith tissue compared with other tissues of the 14 cultivars. A 2-year monitoring assay confirmed the high stable abundance of CLas in fruit pith before the external symptoms were expressed in the fruit rind. We also found the CLas was unevenly distributed within individual fruit pith and tended to colonize in the middle or distal region. In addition, the genome-wide transcriptome profiling of CLas in fruit pith, as well as the anatomical changes of fruit pith caused by CLas infection and the morphology characterization of CLas in fruit pith tissue, was also analyzed. The ability to support multiplication of CLas to high levels by fruit pith makes it an ideal host tissue for morphological studies and *in planta* bacterial transcriptome analyses of CLas–host interaction.

## Materials and Methods

### Plant Materials and DNA Extraction

For the spatial distribution analysis of CLas, the HLB-affected citrus branches of 14 commercial citrus cultivars were collected at fruit maturity season in three provinces (Guangdong, Guangxi, and Zhejiang) in China during November 2018 to January 2019 (Table 1 and Supplementary Figures 1, 2). They were “Pink” grapefruit (*Citrus* × *paradisi* “Pink”), “Eureka” lemon (*C. limon “*Eureka”), “Hu” pomelo (*C. maxima* L. “Changshanhu Yu”), “Shatian” pomelo (“Shatian Yu”), “Liu” sweet orange (*C. sinensis* cv. “Liu Cheng”), “Hongjiang” sweet orange (“Gailiang Cheng”), “Suanju” mandarin (*C. reticulata* Blanco “Suanju”), “Wenzhou” mandarin (“Wenzhou”), “Shatangju” mandarin (“Shatangju”), “Huangyan” mandarin (“Subcompress”), “Nanfeng” mandarin (“Kinokuni”), “Wokan” mandarin (“Wokan”), “Jiaokan” mandarin (“Tankan”), and “Gongkan” mandarin (“Gongkan”). Each citrus branch contained at least one fruit and one new flush (Supplementary Figure 1). For each citrus cultivar, 8–12 symptomatic branches were collected from CLas-infected trees. Samples used for analyses of CLas distribution in the individual citrus branch were collected from three parts, i.e., the new flush, fruit part, and mature leaf (Supplementary Figure 3). For new flush and mature leaves, the leaf midribs were collected for DNA extraction. For branches of “Pink” grapefruit and “Hu” pomelo (Supplementary Figure 1), the mature leaf was not commonly found in HLB-affected branches, and stem bark tissue was collected accordingly. For fruit, three types of subsamples were collected, including the fruit pith, peduncle, and central axis (Supplementary Figure 3). In addition, leaves adjacent to the fruit were also collected for CLas quantification (Supplementary Figure 3).

**TABLE 1:** Quantification of “*Candidatus Liberibacter asiaticus*” (CLas) from six different parts of huanglongbing-affected citrus branches from 14 citrus cultivars.

**No.**	**Cultivar**	**Location**	**Age of host trees (years)**	**No. of citrus branch**	**CLas cells/ng of total DNA^1^**					
					**Leaf midrib (new flush)**	**Pith (fruit)**	**Peduncle (fruit)**	**Central axis (fruit)**	**Leaf midrib (adjacent to fruit)**	**Leaf midrib (mature leaves) or stem bark^2^**
1	*Citrus* × *paradisi* “Pink”	Zhejiang	Seven	10	484 ± 78 b	10,772 ± 2,770 a	218 ± 30 b	7,517 ± 1,331 a	804 ± 429 b	160 ± 47 b
2	*C. limon* “Eureka”	Zhejiang	Five	8	592 ± 116 b	7,690 ± 1,724 a	265 ± 41 b	1,853 ± 610 b	477 ± 92 b	350 ± 115 b
3	*C. maxima* “Changshanhu Yu”	Zhejiang	Eight	10	116 ± 41 b	77,584 ± 17,698 a	2,120 ± 448 b	58,805 ± 13,245 a	972 ± 257 b	160 ± 44 b
4	*C. maxima* “Shatian Yu”	Guangxi	Five	10	610 ± 215 b	12,934 ± 3,688 a	629 ± 135 b	7,123 ± 3,405 a	227 ± 36 b	180 ± 131 b
5	*C. sinensis* “Liu Cheng”	Guangdong	Ten	10	900 ± 300 b	31,026 ± 16,361 a	391 ± 148 b	2,872 ± 856 b	355 ± 169 b	285 ± 258 b
6	*C. sinensis* “Gailiang Cheng”	Guangdong	Six	10	675 ± 354 c	47,537 ± 35,484 a	543 ± 188 c	6,650 ± 2,155 b	492 ± 160 c	85 ± 25 c
7	*C. reticulata Blanco* “Suanju”	Zhejiang	Six	12	1,361 ± 326 c	27,424 ± 3,780 a	756 ± 195 c	8,218 ± 1,562 b	1,963 ± 325 c	509 ± 79 c
8	*C. reticulata Blanco* “Wenzhou”	Zhejiang	Six	12	94 ± 60 b	68,361 ± 20,863 a	172 ± 37 b	10,708 ± 3,802 b	106 ± 51 b	52 ± 24 b
9	*C. reticulata Blanco* “Shatangju”	Guangdong	Seven	10	127 ± 65 b	20,460 ± 4,731 a	2,239 ± 1,281 b	1,813 ± 518 b	194 ± 60 b	195 ± 63 b
10	*C. reticulata Blanco* “Subcompress”	Zhejiang	Five	8	199 ± 95 b	8,143 ± 3,810 a	204 ± 58 b	1,269 ± 579 b	83 ± 56 b	33 ± 11 b
11	*C. reticulata Blanco* “Kinokuni”	Zhejiang	Six	10	93 ± 35 b	9,544 ± 1,599 a	367 ± 103 b	415 ± 143 b	54 ± 33 b	7 ± 2 b
12	*C. reticulata Blanco* “Wokan”	Guangxi	Four	10	85 ± 33 b	20,646 ± 6,424 a	120 ± 55 b	3,369 ± 872 b	62 ± 23 b	35 ± 9 b
13	*C. reticulata Blanco* “Tankan”	Guangdong	Five	10	595 ± 84 c	5,701 ± 1,098 a	463 ± 76 c	2,256 ± 643 b	519 ± 83 c	62 ± 20 c
14	*C. reticulata Blanco* “Gongkan”	Guangdong	Five	8	418 ± 295 b	9,331 ± 4,637 a	109 ± 46 b	1,625 ± 880 b	353 ± 253 b	221 ± 113 b
		Location		138	465 ± 60 c	27,157 ± 4,084 a	633 ± 115 c	8,613 ± 1,622 b	507 ± 69 c	168 ± 27 c

*^1^Single-factor analysis of variance (Duncan multiple-range test) at 95% (*P* = 0.05) confidence interval was used to determine statistical significance. The same line with different letters represents significant difference of CLas density in different parts of citrus branches from individual cultivars. ^2^For “Pink” grapefruit (*Citrus* × *paradisi* “Pink”) and “Hu” pomelo (*C. maxima* “Changshanhu Yu”), no mature leaf was found in the branch, and the stem bark tissue was sampled as alternative.*

For the temporal dynamics analysis of CLas, 10 HLB-affected 4-year-old “Shatangju” mandarin trees from a commercial citrus orchard located in Huizhou city of Guangdong province were selected. For each tree, one citrus branch, with at least one new shoot and one fruit, was collected monthly (from 2017 to 2019). Six different types of tissues (Supplementary Figure 3) were collected from each diseased citrus branch. To obtain adequate samples of fruit for sampling of fruit pith, sampling commenced in August and continued until the end of January (the harvest month) of the next year.

For all samples, 100 mg of flesh tissue was cut into sections of approximately 1 mm wide and ground with an MP FastPrep^®^ −24 Grinder (MP Biomedicals LLC, Santa Ana, CA, United States) under speed of 4 M/S for 1 min. Total DNA was extracted using an E.Z.N.A. HP Plant DNA Kit (OMEGA Bio-Tek Co., Guangdong, China) according to the manufacturer’s manual. The concentration of all extracted DNA samples was determined using Qubit 2.0 (Thermo Fisher Scientific Inc., Waltham, MA, United States).

### Sampling of Fruit Piths

The CLas-infected fruits of three citrus varieties (“Pink” grapefruit, “Wenzhou” mandarin, and “Eureka” lemon), which had relatively large fruits for sampling of long fruit piths, were selected for analyzing the distribution of CLas in fruit pith. For each variety, a total of 10 diseased fruits from different HLB-affected trees were collected. Individual fruit piths were carefully removed intact from HLB-affected fruits. For each fruit, three to five fruit piths were initially pulled out with tweezers, and the longest one (≥6 cm) was selected for further quantification of CLas. Each fruit pith was oriented from calyx-end to stylar-end and cut into four (6 cm ≤ total length < 7 cm) or five (7.5 cm ≤ total length < 8 cm) 1.5-cm segments. Each segment was separately ground with MP FastPrep^®^ −24 Grinder (MP Biomedicals LLC) and used for DNA extraction and CLas quantification analysis.

### Quantification of *Candidatus* Liberibacter asiaticus

The quantitative real-time PCR assays for CLas were performed with primer set (CLas4G/HLBr) and probe (HLBp) according to a previous study (Bao et al., 2020). The standard equation (y = −2.883x + 38.32) (*R^2^* = 0.9995) for quantification of CLas was developed. Briefly, a recombinant plasmid contained the CLas-4G/HLBr primer region was used for construction of the standard equation. The concentration of recombinant plasmid and all DNA extracts were determined using Qubit 2.0 (Thermo Fisher Scientific Inc.). The copy number of plasmid was calculated according to the following formula: the number of copies = (amount in nanograms × Avogadro number)/(length in base pairs × 1 × 10^9^ × 650). The quantification of CLas for each sample was presented as CLas cells per nanograms of total DNA. All TaqMan quantitative real-time PCR was performed in CFX Connect Real-Time System (Bio-Rad, Hercules, CA, United States). The 20 μL of PCR mixture contained 1 μL of DNA template (∼25 ng), 10 μL of Bestar qPCR Master Mix (DBI Bioscience, Shanghai, China), 0.2 μL of PCR Probe (10 μM), 0.4 μL of each forward and reverse primer (10 μM), and 8 μL of ddH_2_O. All PCR was performed under the following procedure: 95°C for 2 min, followed by 40 cycles at 95°C for 10 s and 58°C for 30 s, with fluorescence signal capture at the end of each 58°C step. The data were analyzed using Bio-Rad CFX Manager 2.1 software with automated baseline settings and threshold. The concentration of CLas among different types of tissues from the same cultivar was analyzed by independent-sample *t* test under the SPSS statistical package (v19.0, IBM, Armonk, NY, United States).

### Dual RNA-Seq and Transcriptome Analyses

The high levels of CLas population in fruit pith provided an opportunity to analyze the genome-wide transcriptome profiling of CLas by dual RNA-seq. Therefore, the *in planta* transcriptome analyses of CLas genes in fruit pith and leaf midribs samples were analyzed and compared. The HLB symptomatic leaf samples and fruit pith tissues of the “red-nose” fruit from the same HLB-affected “Shatangju” mandarin branch were collected in December 2018 and then immediately put into the liquid nitrogen before being taken to the laboratory for RNA extraction. Three biological replicates of leaf midribs samples (average Ct value ≈ 22 by primer set of CLas4G/HLBr) and fruit piths samples (average Ct value ≈ 19 by primer set CLas4G/HLBr) were collected. Total RNA was extracted using E.Z.N.A. Total RNA Kit I (OMEGA Bio-Tek Co.) following the manufacturer’s manual. The quality of total RNA sample was tested by Qubit 2.0 (Thermo Fisher Scientific Inc.) and Agilent 2100 (Agilent Technologies Inc., Santa Clara, CA, United States). Library preparation for dual RNA-seq was performed with a TruSeq RNA library Prep Kit (Illumina, San Diego, CA, United States) by removing rRNA from total RNA. High-throughput sequencing was carried out on an Illumina HiSeq 3000 system with 150-bp paired-end reads by a commercial sequencing company.

For CLas transcriptome analysis, all clean HiSeq data from leaf midribs and fruit piths samples were mapped to CLas strain A4 genome (CP010804.2) by CLC Genomic Workbench v9.5 (Qiagen Bioinformatics, Aarhus, Denmark) (length faction = 0.95; similarity fraction = 0.95). Reads mapped to each CLas gene were then summarized into count tables of “Total Gene Reads.” The normalization of RNA-seq was referenced to the transcripts per kilobase million (TPM) method, i.e., TPM = *A* × 10^6^ × 1/Σ(*A*), where *A* = total reads mapped to gene × 10^3^/gene length in bp. Differentially expressed genes (DEGs) between leaf midribs HiSeq data and fruit pith HiSeq data were performed with GFOLD V1.1.4 (Feng et al., 2012). Log2 fold change ≥ | 1 | was set as cutoff values. All identified DEGs were further submitted for functional annotation and ortholog assignment with eggNOG mapper (Huerta-Cepas et al., 2017). The top 20 most highly expressed genes evaluated by TPM value from leaf midribs data and fruit pith data were manually retrieved. Heatmap for comparison of CLas gene expression (by TPM value) between leaf midribs and fruit pith was generated in TBtools software (Chen et al., 2020).

For host transcriptome analyses, all clean HiSeq data from leaf midribs and fruit piths samples were mapped to *Citrus clementina* v1.0 reference genome (Wu et al., 2014) by using Tophat2 with the mismatch penalty of no more than two nucleotides (Kim et al., 2013). DEGs between leaf midribs and fruit pith were identified with DEGseq (Wang et al., 2010). Log2 fold change ≥ | 1 | and *q* < 0.005 were set as cutoff values. DEGs were assigned into GO (Gene Ontology) categories with GOseq (Young et al., 2010). Kyoto Encyclopedia of Genes and Genomes (KEGG) pathway enrichment analysis of DEGs was conducted using KOBAS 2.0 software (Xie et al., 2011).

### Light Microscopy and Transmission Electron Microscopy

Leaf midribs and fruit pith tissues from both HLB-affected and healthy “Gongkan” and “Shatangju” mandarin trees were used for light microscopy assessments. All tissues were initially cut into approximately 3-mm segments and further fixed with 3% glutaraldehyde in 0.1 M potassium phosphate buffer (pH 7.2) at 4°C overnight. The fixed tissues were then washed in the same buffer and postfixed in 2% osmium tetroxide for 4 h at room temperature, dehydrated in an acetone series, and embedded in Spurr resin. One micrometer of sections was cut with glass knives and stained with safranin O and fast green solution. The stained tissues were dehydrated and cleared with 95% ethyl alcohol, absolute alcohol, and xylene using two changes each (2 min each). Light micrographs were taken on a Nikon Eclipse Ni microscope (Nikon Instruments Inc., Melville, NY, United States) with a Nikon DS-Ri2 camera. The fruit pith tissues with high abundance of CLas were used for transmission electron microscopy (TEM) analyses. For TEM, 100-nm sections of CLas-infected fruit pith tissues were cut with a diamond knife, stained with 2% aq. uranyl acetate, and poststained with lead citrate. TEM micrographs were taken by an AMT (Advanced Microscopy Techniques Corp., Danvers, MA, United States) digital camera on a Morgagni 268 (FEI Company, Hillsboro, OR, United States) transmission electron microscope.

## Results

### Distribution of CLas in Citrus Branches and Fruits of 14 Citrus Cultivars

Quantification analyses showed CLas was most abundant in the fruit pith (27,157 cells/ng of total DNA), followed by central axis (8,613 cells/ng of total DNA), peduncle (633 cells/ng of total DNA), midribs of leaf adjacent to fruit (507 cells/ng of total DNA), leaf midribs of new flush (465 cells/ng of total DNA), and midribs of mature leaf (168 cells/ng of total DNA). Statistical analysis revealed that the concentration of CLas was significantly higher in fruit pith (11 citrus cultivars) or in both fruit pith and central axis than in other tissues collected from the same citrus branch (*P* < 0.05) (Table 1). The concentration of CLas in fruit pith varied among 14 citrus varieties, ranging from 5,701 cells/ng of total DNA (“Tankan” mandarin) to 77,584 cells/ng of total DNA (“Shatian” pomelo) (Table 1). In addition, the lowest concentrations of CLas were mainly recorded in the midribs of mature leaf than other tissues in 14 citrus varieties (Table 1).

### Temporal Dynamics of CLas Population in HLB-Affected “Shatangju” Mandarin Branches

**FIGURE 1:**
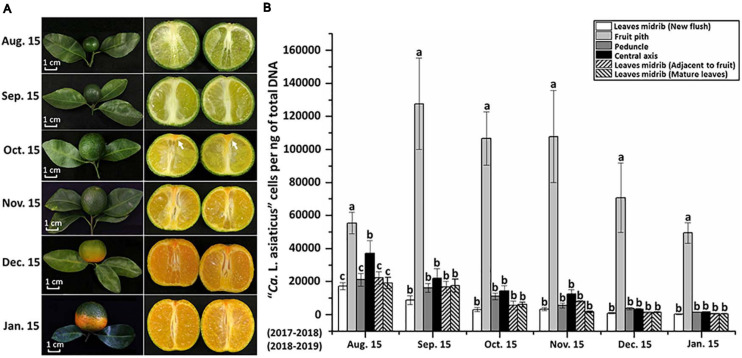
Quantitative distribution off “*Candidatus*f Liberibacter asiaticus” (CLas) in citrus branches and fruits. **(A)** Symptom development of HLB-affected “Shatangju” mandarin (*Citrus reticulata* Blanco “Shatangju”) fruits. **(B)** Temporal dynamics of CLas population in different parts of HLB-affected “Shatangju” branches. Ten citrus branches were monthly collected from HLB-affected citrus trees during August 15, 2017, to January 15, 2018, and August 15, 2018 to January 15, 2019. Note the vascular columella (white arrow) under the peduncle of diseased fruit started to turn into orange in October.

The temporal dynamics analyses of CLas population showed that CLas concentration in fruit pith was consistently higher than other parts in “Shatangju” branches collected monthly from August to January (*P* < 0.05) (Figure 1B). The CLas populations peaked in September and then decreased slightly as fruit ripened (in December and January) (Figure 1B). However, although the highest concentrations of CLas were detected in fruit pith collected in September, “red-nose” symptom was not observed in HLB-affected “Shatangju” fruits in September (Figure 1A). In contrast, typical “red-nose” symptom was observed in diseased fruits collected at January, when CLas concentrations in fruit pith were at a lowest level recorded over 6 months (Figure 1B). Concentrations of CLas in the other tissues sampled also declined gradually from peaks in August to January when the lowest concentrations were recorded (Figure 1B).

### Quantification and Distribution of CLas in Fruit Pith of Three Citrus Cultivars

**FIGURE 2:**
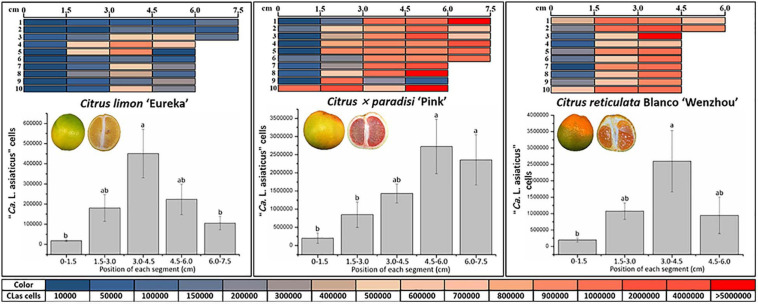
Quantitative distribution of “*Candidatus* Liberibacter asiaticus” (CLas) in fruit piths of three citrus varieties. Graphic denoted 1.5-cm segment of fruit piths. Color scale indicates the CLas cell number in each 1.5-cm segment following quantitative real-time PCR. The average number of CLas cells in each 1.5-cm segment of fruit piths from individual citrus variety is indicated as bar graph. The different letters at the top of the bar represent significant difference by single-factor analysis of variance (Duncan multiple-range test) at 95% (*P* < 0.05) confidence interval.

Overall, the average population of CLas in 1.5-cm segment of “Eureka” lemon pith (209,916 ± 42,937) was significantly lower than in those from “Pink” grapefruit (1,418,281 ± 241,315) and “Wenzhou” mandarin (1,261,757.66 ± 334,300) (*P* < 0.05) (Figure 2). CLas population ranged from 8.6 × 10^3^ to 1.3 × 10^7^, 2.0 × 10^3^ to 9.1 × 10^6^, and 5.8 × 10^3^ to 1.1 × 10^6^ cells for each of the 1.5-cm pith segment from “Wenzhou” mandarin, “Pink” grapefruit, and “Eureka” lemon, respectively (Figure 2). Fresh 1.5-cm segment of pith weighted, on average, approximately 8 mg. Therefore, CLas populations ranged from 1.0 × 10^6^ to 1.6 × 10^9^, 2.5 × 10^5^ to 1.1 × 10^9^, and 7.3 × 10^5^ to 1.4 × 10^8^ cells per gram of fresh fruit pith from “Wenzhou” mandarin, “Pink” grapefruit, and “Eureka” lemon, respectively.

Quantitative analyses revealed CLas was unevenly distributed in fruit pith (Figure 2). In “Eureka” lemon fruit, the population of CLas was significantly higher in the 1.5-cm segments of pith sampled from 3.0 to 4.5 cm in the middle region than those from stylar and calyx region (from 0 to 3.0 cm and 4.5 to 7.5 cm) (Figure 2). In fruit of “Pink” grapefruit and “Wenzhou” mandarin, the significantly higher CLas population was observed in the 1.5-cm segments of pith sampled from the stylar region (from 4.5 to 7.5 cm of “Pink” grapefruit and from 3.0 to 4.5 cm “Wenzhou” Mandarin) than in the 1.5-cm segments of calyx region (from 0 to 3.0 cm of “Pink” grapefruit and “Wenzhou” mandarin) (Figure 2).

### Genome-Wide Gene Expression Profiling of CLas in Leaf Midribs and Fruit Piths

**FIGURE 3:**
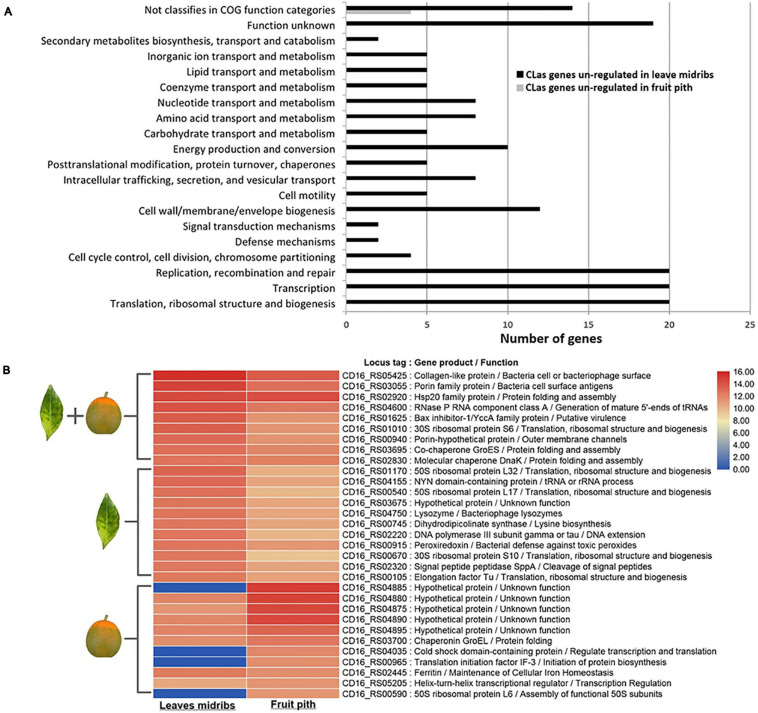
Functional classification of differentially expressed genes **(A)** and top 20 highly expressed gene **(B)** of “*Candidatus* Liberibacter asiaticus” (CLas) in fruit pith and leaf midribs. The differentially expression genes (DEGs) are identified with GFOLD v1.1.4 software by setting log2 fold change ≥ | 1 |. Classification of DEGs is performed with EggNOG v5.0 by using Clusters of Orthologous Groups database. Black bar represents genes up-regulated in fruit pith compared with leaf midribs. Gray bar represents genes up-regulated in leaf midribs compared with fruit pith. Heatmap shows the log2 of normalized TPM values for the top 20 most highly expressed CLas genes in leaf midribs tissue and fruit pith tissue. Genes that belong to the list of the top 20 most highly expressed genes in leaf midribs or fruit piths are labeled with leaf or fruit symbol in the left.

Approximately 100 and 147 million HiSeq reads were obtained from CLas-infected citrus leaf midribs and fruit piths RNA samples, respectively. Mapping to CLas A4 genome (CP010804.2) identified a total of 3,423 reads (average coverage of 0.4 × of mapped consensus) from leaf midribs RNA-seq data and 109,191 reads (average coverage of ∼13 × to the mapped consensus) from fruit piths RNA-seq data. Differential expression analyses revealed 151 DEGs of CLas between leaf midribs and fruit piths. Of 151 DEGs, 147 were significantly up-regulated (log2 fold change ≥ | 1 |) in leaf midribs compared with fruit piths, whereas only four CLas genes were significantly up-regulated in fruit piths compared with leaf midribs (Supplementary Table 1). Functional classification showed 151 DEGs were categorized into 19 diverse function groups plus one group of genes not classified in the COG database (Figure 3A). Large numbers of genes involved in replication, recombination, repair, transcription, translation, transport/metabolism, and cell wall/membrane/envelope biogenesis were up-regulated in leaf midribs compared with fruit pith tissue (Figure 3A).

It was found that 10 genes involved in active transport system were up-regulated in leaf midribs compared with fruit pith (Supplementary Table 1), including five ABC transporter genes (CD16_RS00210, CD16_RS00215, CD16_RS02450, CD16_RS03160, and CD16_RS04765), one glycine betaine transporter gene (CD16_RS01075), and four genes involved in the efflux pumps system (CD16_RS00510, CD16_RS03560, CD16_RS04390, and CD16_RS05425) (Supplementary Table 1). The expression of genes involved in flagellar assembly, including *flaF* (CD16_RS03425), *flgH* (CD16_RS01235), *flgA* (CD16_RS01250), *flgK* (CD16_RS03415), and *fliK* (CD16_RS03400), and the CD16_RS02435 involved in pilus assembly, were also up-regulated in leaf midribs (Supplementary Table 1). It should be noted that genes involved in virulence/toxicity were overexpressed in leaf midribs tissue and included three genes involved in lipid A or lipopolysaccharide (LPS) biosynthesis (CD16_RS01575, CD16_RS02310, CD16_RS03055, and CD16_RS05315) and CD16_RS01040 involved in toxin biosynthetic process (Supplementary Table 1). In addition, *gndA* (CD16_RS02465) and *gshB* (CD16_RS02570), encoding enzymes involved in the metabolism of glutathione, were also up-regulated in the leaf midribs. However, four CLas genes that up-regulated in fruit pith were not assigned with any COG function categories (Supplementary Table 1).

The top 20 highest expressed CLas genes in leaf midribs and fruit pith were compared (Figure 3B). Three genes involved in bacterial cell surface structure, including two porin family genes (CD16_RS03055 and CD16_RS00940) and a collagen-like protein gene (CD16_RS05425), were highly expressed in both leaf midribs and fruit pith tissue (Figure 3B). A Bax inhibitor-1/YCCA family gene (CD16_RS01625), encoded putative plant cell death suppressor, was also highly expressed in leaf midribs and fruit pith tissue (Figure 3B). Three known stress-related genes, *DnaK* (CD16_RS02830), *GroES* (CD16_RS03695), and *GroEL* (CD16_RS03700), and a heat shock protein (CD16_RS02920) were found to express in a high level in leaf midrib and fruit pith (Figure 3B). In addition, the high expression level of a peroxiredoxin gene (CD16_RS00915) was also detected in leaf midrib and fruit pith tissue.

### Transcriptome Profiling of Leaf Midribs and Fruit Pith Response to CLas Infection

**FIGURE 4:**
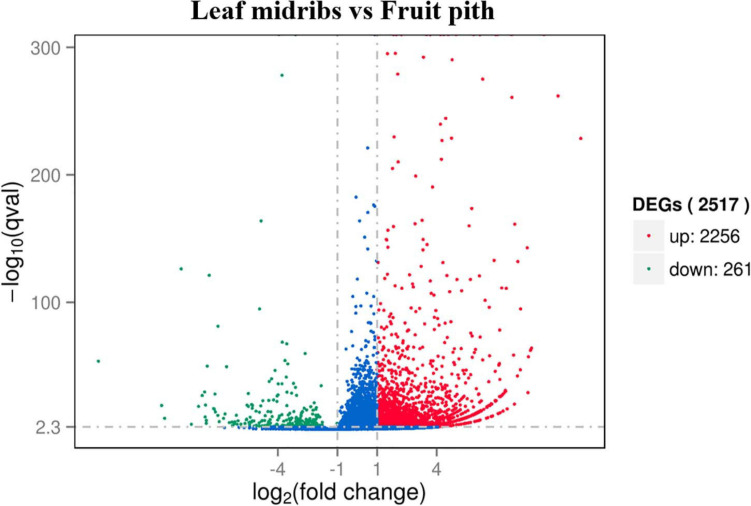
Volcano plot representation of differential expression analysis of host genes in “*Candidatus* Liberibacter asiaticus” (CLas)–infected leaf midribs and CLas-infected fruit pith. Red and green points mark the genes with significantly increased or decreased expression, respectively, in leaf midribs compared with fruit pith samples (FDR < 0.01). The blue points mark the genes with no significantly different expression. The *x*-axis represents log2 fold changes in expression, and the *y*-axis represents statistically significant difference in gene expression.

**FIGURE 5:**
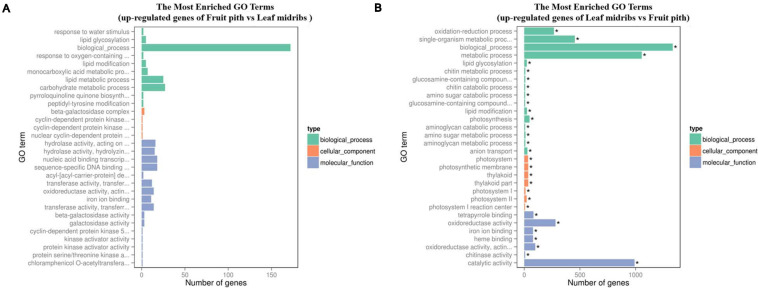
GO term enrichment analyses of the up-regulated genes in “*Candidatus* Liberibacter asiaticus” (CLas)–infected fruit pith **(A)** and CLas-infected leaf midribs **(B)**. The vertical coordinates represent enriched GO terms, and the horizontal coordinates represent the numbers of up-regulated genes in these GO terms. Green columns: biological process GO terms; purple columns: cellular component GO terms; orange columns: molecular function GO terms. *Significantly enriched GO term.

**FIGURE 6:**
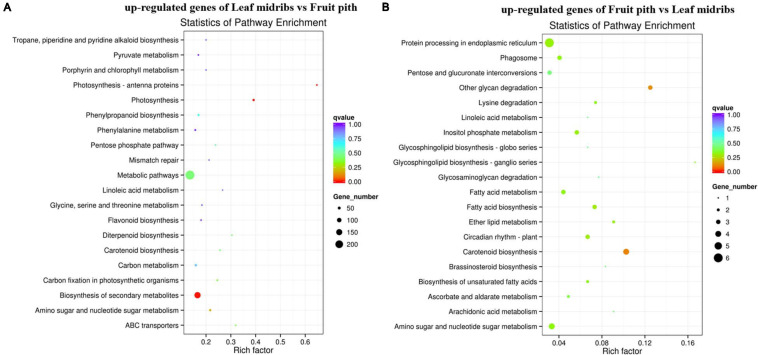
KEGG enrichment analyses of the up-regulated genes in “*Candidatus* Liberibacter asiaticus” (CLas)–infected leaf midribs **(A)** and CLas-infected fruit pith **(B)**. The vertical coordinates represent the enriched pathways, and the horizontal coordinates represent the rich factors. The size of each point represents the number of up-regulated genes in the pathway, and the color of the point represents the *q* value.

Approximately 92.67% of leaf midribs HiSeq reads and 97.42% of fruit pith HiSeq reads were successfully mapped to *C. clementina* genome. A total of 2,517 DEGs were identified between CLas-infected leaf midribs and fruit pith. There were 2,256 DEGs up-regulated in CLas-infected leaf midrib, whereas 261 DEGs were up-regulated in CLas-infected fruit pith (Figure 4). GO enrichment analysis showed that the up-regulated DEGs in CLas-infected leaf midribs were mainly enriched in biological process, metabolic process, catalytic activity, single-organism metabolic process, oxidation–reduction process and oxidoreductase activity, whereas the up-regulated DEGs in CLas-infected fruit pith were mainly involved in biological process (Figure 5). Among genes enriched in biological process, a gene-encoded leucine-rich repeat receptor-like protein kinase (Ciclev10024853m.v1.0) was strongly up-regulated in CLas-infected leaf midribs compared with CLas-infected fruit pith (log2 fold change = 8.21) (Supplementary Table 2). In addition, the result of KEGG pathway analysis revealed that the large numbers of up-regulated DEGs in CLas-infected leaf midribs were mainly enriched in metabolic pathway and biosynthesis of secondary metabolites (Figure 6). The pathway of biosynthesis of secondary metabolites mainly contained genes involved in the synthesis of secondary metabolites, which have functions such as antimicrobial activities, including acridone, germanicol, chalcone, cinnamyl alcohol, neomenthol, caffeic acid, flavonol, β-amyrin, and chitinase (Supplementary Table 3). In contrast, genes involved in protein processing in endoplasmic reticulum, carotenoid biosynthesis, amino, and nucleotide sugar metabolism were up-regulated in CLas-infected fruit pith.

### Anatomical Changes in the Phloem of Leaf Midribs and Fruit Piths Caused by CLas Infection

**FIGURE 7:**
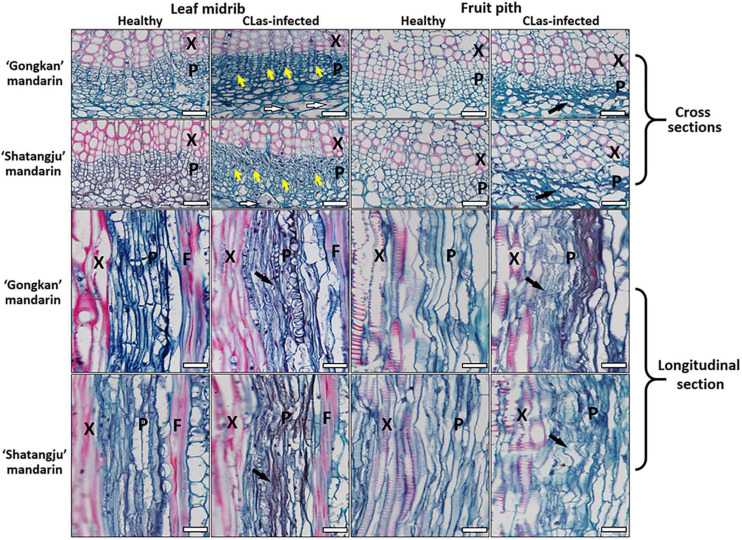
Light micrographs of leaf midribs and fruit pith from “*Candidatus* Liberibacter asiaticus”–infected and healthy “Gongkan” and “Shatangju” mandarin. X, xylem; F, fiber; P, phloem. The callose plugs (yellow arrow), collapsed phloem (black arrow), and starch granule accumulation (white arrow) are commonly observed in CLas-infected lead midrib. Bar = 30 μm.

Although the CLas cells were abundant in fruit pith tissues, the microscopic manifestations of fruit pith caused by CLas infection were unclear. Compared with the healthy citrus, the phloem of CLas-infected fruit pith was severely collapsed with cell wall distortion (Figure 7, black arrow), especially the sieve elements that appeared irregular with jagged boundaries (Figure 7, black arrow). The thickened and disrupted cell walls of the sieve element and companion cells were also commonly found in the phloem of CLas-infected fruit piths (Figure 7). Compared with the CLas-infected leaf midribs tissue, a higher level of phloem collapse was observed in CLas-infected fruit pith (Figure 7). In contrast, a relatively higher concentration of starch granule accumulation was observed in parenchyma cells of CLas-infected leaf midribs, whereas no starch accumulation was observed in CLas-infected fruit pith (Figure 7).

**FIGURE 8:**
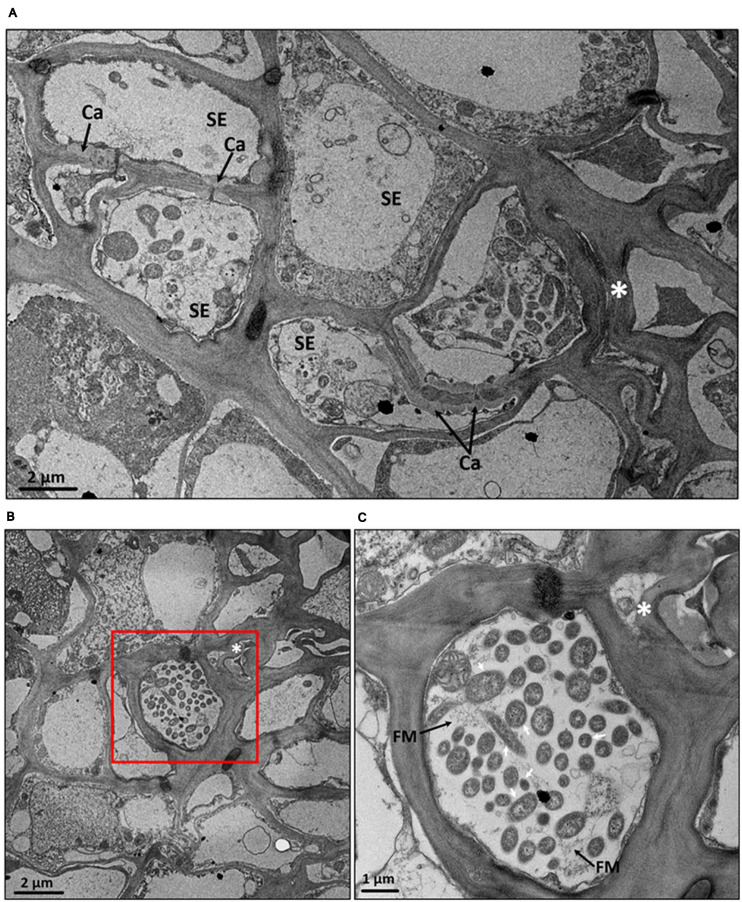
Transmission electron micrographs of cross section of fruit pith tissues from “*Candidatus* Liberibacter asiaticus”–infected “Shatangju” mandarin. **(A)** Collapsed phloem cells and callose deposition in the plasmodesmata pore between sieve elements of CLas-infected fruit pith. **(B)** A high abundance of CLas cells with elongated and round forms in the phloem cell. **(C)** The close-up of red region in panel **(B)**. SE, sieve element; Ca, callose; FM, filamentous material found around CLas cells. *Phloem cells collapse. Note double cell walls (white arrows) characteristic of Gram-negative bacterium, which is presumed to be CLas cells.

The severe collapse with heavy callose deposition around the plasmodesmata pore of sieve element in phloem of CLas-infected fruit pith was also confirmed by the TEM analysis (Figure 8A). Two possible forms of CLas cells, the elongated and round forms, were present at a high abundance in phloem cells of CLas-infected fruit pith (Figures 8B,C). A high magnification electron of TEM micrographs revealed the unknown filamentous materials were commonly found around CLas cells and connected between CLas cells (Figure 8C).

## Discussion

The current inability to culture CLas *in vitro* leads to the characterization of CLas need to rely on the analysis of CLas-abundant host tissue. In this study, we found a stable high abundance of CLas in fruit pith of 14 citrus cultivars. Previous studies have reported relatively high concentrations of CLas in fruit tissue, for instance, in the peduncles (Tatineni et al., 2008) and locular membranes/septa (Li et al., 2009). As phloem-limited bacteria, CLas could be able to move with phloem flow from source tissues (e.g., mature leaves) to sink tissues (e.g., fruit) and accumulated in higher numbers in the sink tissue (Tatineni et al., 2008; Achor et al., 2020). The pith of citrus fruit used in this study mainly comprises the scattered dorsal vascular bundles (Supplementary Figure 3), which transport nutrients for fruit developing. Therefore, the high level of CLas population in fruit pith tissue was mainly because it can be acted as the nutritional sink for CLas accumulation and contained the abundance of nutrients for CLas multiplication. This also implies that fruit pith tissue can be used as ideal host material for CLas multiplication. Further analyses of nutrients composition and microenvironment in the fruit pith can help to identify fastidious requirements for *in vitro* culturing of CLas. Nonetheless, the density of CLas populations in the fruit pith tissue from different citrus cultivars varied, ranging from 5,701 to 77,584 cells/ng of total DNA (Table 1). The different nutritional contents, metabolites, or even resistance substances in the phloem sap of different citrus cultivars could have influence on CLas populations in fruit piths among different cultivars. Therefore, further comparison of phloem sap composition of fruit pith from different citrus cultivars should help to identify substances responsible for tolerance/susceptibility to CLas, as well as the essential requirements for culturing CLas *in vitro*.

Quantification analyses of CLas populations in consecutive 1.5-cm length of fruit pith between the calyx (proximal) and stylar (distal) regions of mature fruit of three citrus cultivars showed that CLas cells were unevenly distributed in fruit pith, with populations in central and stylar region higher than in the calyx region (Figure 2). The fruit pith collected in this study mainly comprised dorsal vascular bundles, which was part of the vascular system of citrus fruit. The vascular system of citrus fruit forms a broadly branched network of main vascular bundles and subsidiary traces, including the axial, dorsal, septal, and marginal bundles (Tadeo et al., 2020). In citrus fruit, the central axial bundle extends down to the stylar end, diverges into the septa to become the marginal bundles, then merges with the dorsal and septal bundles at the stylar end, and finally closes the vascular circuit at this region (Ladanyia, 2010; Tadeo et al., 2020). Consequently, the distribution of photosynthates and sugars is higher in the stylar region than the calyx/stem region (Ladanyia, 2010). Therefore, the higher populations of CLas cells in the middle and stylar region than in the calyx region could be attributed to the distribution of nutrients.

Details on genome-wide transcriptome profile of CLas in infected citrus trees have not been described yet mainly due to the low abundance of CLas RNA compared with host citrus RNA in total RNA samples. Compared with the leaf midrib sample, the use of CLas-abundant fruit pith samples for dual RNA-seq generated a higher depth (>10×) of CLas genome and allowed more genes to be quantified, indicating a higher resolution of transcriptome profile for CLas revealed by using CLas-infected fruit pith. It was found that four CLas genes highly expressed in fruit pith were not detectable in leaf midribs by HiSeq data (Figure 3B). However, annotation showed three of four genes (CD16_RS04035, CD16_RS00965, and CD16_RS00590) were involved in the essential function of transcription and translation process of CLas (Figure 3B), indicating the undetectable expression of these constitutive genes could be due to the shallow sequencing depth of CLas genes in RNA-seq of leaf midribs sample.

Differential gene expression analyses still identified a total of 151 DEGs, with most (147 DEGs) of them up-regulated in leaf midrib compared with fruit pith. The higher number of up-regulated CLas genes in leaf midribs implied the CLas–host interaction could be much stronger in leaf midribs than in fruit pith. In addition, nearly a third of CLas DEGs that were mainly involved in the replication, transcription, translation, ribosomal structure, and biogenesis process were up-regulated in leaf midribs compared with fruit pith (Figure 3A). This suggested that CLas population could be still under the growth phase in the leaf midribs in December. By contrast, the inactive CLas gene expression and the starting decline of CLas population in December (Figure 1B) suggested the CLas population could be in a decline/death phase in the fruit pith collected in December.

Genes involved in active transport system of CLas were up-regulated in leaf midrib compared with fruit pith. ABC transporters are known to be involved in bacteria virulence associated with the uptake of nutrients (Boyer et al., 2002). The overexpression of five genes involved in ABC transporters in leaf midribs suggested they could be involved in virulence of CLas in leaf. Four genes associated with the efflux pumps system were also up-regulated in leaf midribs (Supplementary Table 1). The bacterial efflux pumps were able to extrude a wide range of toxic compounds out of cells, such as antibiotic, heavy metals, organic pollutants, plant-produced compounds, quorum sensing signals, or bacterial mechanism (Blanco et al., 2016). In addition, genes (*gndA* and *gshB*) involved in the metabolism of glutathione were also found to be up-regulated in leaf midrib (Supplementary Table 1). The bacterial glutathione peroxidase was known as a common antioxidant in protecting bacterial cells against oxidative stress (Arenas et al., 2011). The up-regulation of efflux pump genes and stress resistance–related genes in leaf is believed to protect CLas from harmful plant environments in leaf, which also indicated that CLas could be under a higher plant toxins or defense responses in leaf midrib compared with fruit pith.

The up-regulated CLas LPS genes in leaf midribs may play a critical role in CLas–host interaction. In this study, four CLas LPS genes were up-regulated in leaf midribs (Supplementary Table 1). The up-regulation of LPS genes could be important for CLas to survive in leaf, as LPS is the major component of the outer membrane in Gram-negative bacteria and can be served as a physical barrier to protect bacterial cells from the unfavorable host plant environments (Rosenfeld and Shai, 2006). In addition, the bacterial LPS was known to play an important role in eliciting plant basal defense-related response by acting as a pathogen-associated molecular pattern (PAMP) (Parker, 2003). The bacterial PAMP can be recognized by host innate immunity system during the infection (Parker, 2003). This was consistent with our observation that the gene (Ciclev10024853m.v1.0) that encoded the leucine-rich repeat receptor-like protein, a critical gene in plant innate immunity, was strongly up-regulated in leaf midribs compared with fruit pith (Supplementary Table 2).

Genes involved in CLas cell surface structure, stress response, and virulence was highly expressed in both leaf midribs and fruit pith. A large number of collagen-like proteins have been identified in bacteria and play a critical role in extracellular matrix structural properties and cell signaling. In *Bacillus*, the glycosylated collagen-like proteins have been shown to be present in thin hair-like surface filaments of bacteria cells (Yu et al., 2014). The high expression level of CLas collagen-like gene (CD16_RS05425) in fruit pith tissue was consistent with the observation of filamentous material around CLas cells (Figure 8C), although its function remains to be determined. The BAX inhibitor was known as an ancient conserved cell death suppressor in mammals and plants with prokaryotic relatives (Hückelhoven, 2004). In addition, peroxiredoxin was found to play an important role in bacterial defense against oxidative stress and toxic peroxides (Dubbs and Mongkolsuk, 2007). The high expression of the BAX inhibitor-1/YCCA family gene and peroxiredoxin gene in leaf midribs and fruit pith indicated they might contribute to the compatible interaction between the pathogen and host plant by suppressing of the host cell death and defensing against the oxidative stress and toxic chemicals produced by host, respectively.

The secondary metabolites may play an important role in plant defense against CLas in leaf midribs. We observed that large numbers of genes involved in the synthesis of antimicrobial secondary metabolites were up-regulated in leaf midribs (Figure 6 and Supplementary Table 3). Especially, an acridone synthase gene (Ciclev10025807m.v1.0), germanicol synthase gene (Ciclev10033766m.v1.0), and a chalcone synthase gene (Ciclev10032697m.v1.0) were strongly up-regulated in leaf midribs compared with fruit pith (log2 fold change > 4). The acridone and its derivatives were commonly found in plant and exhibited the antibacterial activity (Wainwright, 2001). Germanicol was found as one of the main constituents in essential oil of the *Pistacia lentiscus* leaves, which showed strong activity against *Klebsiella pneumoniae* (Mharti et al., 2011). Chalcones is one of the important compound groups of flavonoids and exhibited a wide spectrum of biological activities, such as antimicrobial activity (Xu et al., 2019). Therefore, the up-regulated genes involved in secondary metabolites against microbe/bacteria in leaf midribs compared with fruit pith revealed a lower concentration of antimicrobial compound produced by host in fruit pith than in leaf midrib, which could also explain why the fruit pith could be a more suitable host tissue for CLas reproducing.

Anatomical changes in phloem caused by CLas infection mainly included the phloem collapse with jagged and thickened cell walls, hypertrophic parenchyma cells, callose plugging, and massive starch accumulation (Schneider, 1968; Etxeberria et al., 2009; Kim et al., 2009; Achor et al., 2010; Folimonova and Achor, 2010; Koh et al., 2012; Brodersen et al., 2014). Callose deposition induced by CLas infection blocks transport of photoassimilate in phloem. This leads to starch accumulation in phloem (Schneider, 1968; Koh et al., 2012). Phloem collapse and plugging led to the starch packing in chloroplasts of host cells, manifested as chlorosis associated with HLB (Achor et al., 2010). Based on our observation, starch accumulation was not observed in the CLas-infected fruit pith tissue but was common in CLas-infected leaf midribs (Figure 7). Starch grain accumulation in infected citrus was usually observed in the parenchyma tissue, as well as in phloem cells, but they were often concealed by the collapsed phloem cells in phloem tissue (Etxeberria et al., 2009).

Severe collapse of phloem cells and cell wall distortion in CLas-infected fruit pith were more apparent than in leaf midribs (Figure 7). This may have been related to higher abundance of CLas population in fruit pith. Previous studies have indicated that phloem collapse caused by CLas infection is primarily due to hyperactive differentiation of vascular cambium and hyperplasia of parenchyma cells surrounding the necrotic phloem pocket (Kim et al., 2009). Moreover, the significant swelling of middle lamella between cell walls surrounding sieve elements occurred at an early stage of CLas infection, also potentially leading to phloem collapse and necrosis in infected leaves (Folimonova and Achor, 2010). However, although the phloem collapse was identified as an intrinsic characteristic caused by CLas infection, the mechanism of how CLas causes the damage on phloem and the host response in fruit pith tissues was still unclear. It is possible that the CLas might secrete the virulence factors into the phloem, trigger the plant defense, and cause the phloem collapse.

In addition to phloem collapse and starch accumulation, callose deposition in the sieve pores and plasmodesmata is another characteristic ultrastructure change in the CLas-infected lead midrib (Kim et al., 2009; Achor et al., 2010). In this study, we observed that a heavy callose accumulates around the phloem plasmodesmata pore of sieve elements in CLas-infected fruit pith (Figure 8A). The callose plugging in plasmodesmata was also known to be a plant defense response to diverse biotic and abiotic stresses, such as insect/pathogen attack (Nishimura et al., 2003; Rinne and Schoot, 2003; Roberts, 2003), wounding (Radford et al., 1998), and cellular plasmolysis (Drake et al., 1978). A previous study also suggested that the CLas infection triggered production of reactive oxygen species, which in turn promoted callose synthesis at plasmodesmata (Koh et al., 2012).

Based on the high-magnification electron of TEM, we observed two possible forms of CLas cells, elongated and round forms, in phloem cell of fruit pith (Figures 8B,C). Although it was not certain whether both elongated and round forms of cells were CLas, the similar types (both elongated and round shapes) of CLas cells have been observed and confirmed in early studies (Hilf et al., 2013; Achor et al., 2020). Especially, a recent study showed the CLas was able to change its morphology from round form to elongated form, which enables the CLas movement through host phloem pores (Achor et al., 2020). A further immuneolabeling or fluorescence *in situ* hybridization analysis of CLas cell is required. In addition, the filamentous-like material was commonly found around CLas cells and connected between CLas cells (Figure 8C). The filamentous material around CLas cells was thought to be utilized by CLas for attachment of bacterial cells to the host cell membrane adjacent to the phloem pores (Achor et al., 2020). However, no adhesion protein of CLas has been described. Interestingly, a bacterial (or bacteriophage) cell surface–associated gene (CD16_RS05425) that encoded a collagen-like protein was found to be highly expressed in both CLas-infected citrus leaf midribs and fruit piths (Figure 3B). This suggested that this gene may be involved in the CLas–host interaction.

In conclusion, based on the distribution analyses of CLas in citrus branches of 14 citrus cultivars, we found that a significantly high abundance of CLas cells was consistently detected in fruit pith of all citrus cultivars. Highest densities of CLas in fruit pith occurred before visual symptoms of HLB developed in infected fruits. CLas was unevenly distributed within fruit pith and tended to colonize in the middle or stylar region of fruit pith. The use of CLas-abundant fruit pith sample for dual RNA-seq provided the reliable and high-resolution of CLas transcriptome profile compared by using CLas-infected leaf midribs. CLas infection caused the severe phloem collapse and the heavy callose deposition in the plasmodesmata of sieve elements in fruit pith. The highly simplified anatomy of fruit pith and its ability to support multiplication of CLas to high abundance make it as an ideal host material for further studies on the morphology and transcriptomic analyses of CLas–host interaction.

## Data Availability Statement

RNA-seq data from CLas-infected leaf midribs and fruit pith samples supporting the findings of this study are available at the National Center for Biotechnology Information under SRA accession PRJNA660208.

## Author Contributions

FF, HG, MX, XD, and ZZ conceived and designed the experiments. FF, HG, AZ, TL, ZZ, and HL performed the experiments. FF and ZZ contributed to bioinformatics and statistical analysis. FF, HG, and ZZ prepared figures/table and wrote the draft manuscript. MX, XD, and ZZ reviewed the manuscript. All authors contributed to the article and approved the submitted version.

## Conflict of Interest

The authors declare that the research was conducted in the absence of any commercial or financial relationships that could be construed as a potential conflict of interest.
