# Supplementary material for: A Significantly High Abundance of “Candidatus Liberibacter asiaticus” in Citrus Fruit Pith: in planta Transcriptome and Anatomical Analyses
Source: Front Microbiol. 2021 Jun 11;12:681251. doi: 10.3389/fmicb.2021.681251 (PMC8225937; doi:10.3389/fmicb.2021.681251)
Supplement: Supplementary file 1 [file Data_Sheet_1.PDF]

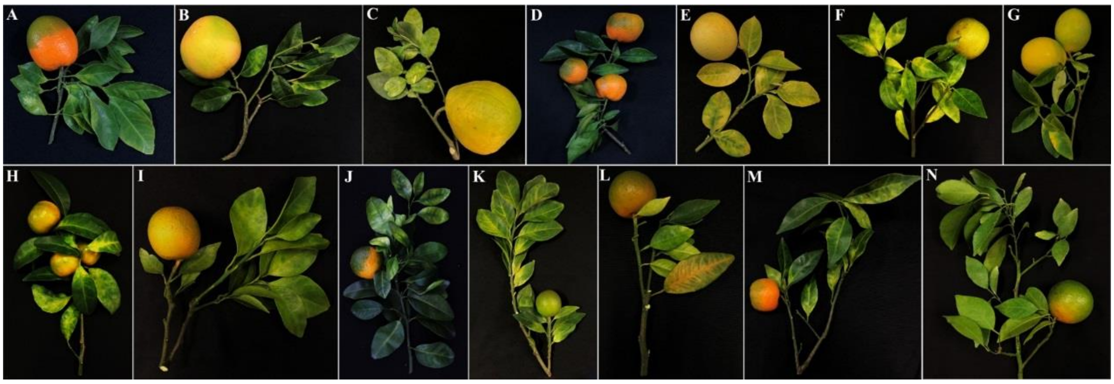

Fig. S1. Symptoms of huanglongbing-affected citrus branches (with fruit) from 14 citrus cultivars. A. *Citrus reticulata Blanco* 'Wenzhou', B. *C.* × *paradisi* 'Pink', C. *C. maxima* 'Shatian Yu'. D. *C. reticulata Blanco* 'Subcompress'. E. *C. maxima* 'Changshanhu Yu'. F. *C. reticulata Blanco* 'Tankan'. G. *C. limon* 'Eureka'. H. *C. reticulata Blanco* 'Suanju'. I. *C. sinensis* 'Liu Cheng'. J. *C. reticulata Blanco* 'Shatangju'. K. *C. sinensis* 'Gailiang Cheng'. L. *C. reticulata Blanco* 'Wokan'. M. *C. reticulata Blanco* 'Kinokuni'. N. *C. reticulata Blanco* 'Gongkan'.

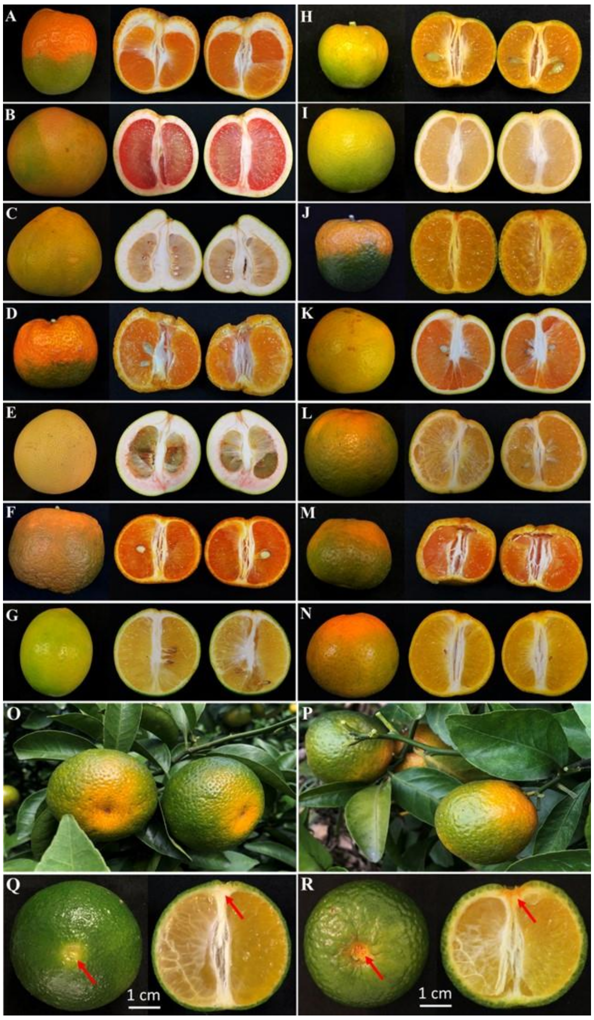

Fig. S2. Symptoms of Huanglongbing-affected citrus fruit from 14 citrus cultivars and healthy citrus fruits. A. *Citrus reticulata Blanco* ‘Wenzhou’, B. *C.* × *paradisi* ‘Pink’, C. *C. maxima* ‘Shatian Yu’. D. *C. reticulata Blanco* ‘Subcompress’. E. *C. maxima* ‘Changshanhu Yu’. F. *C. reticulata Blanco* ‘Tankan’. G. *C. limon* ‘Eureka’. H. *C. reticulata Blanco* ‘Suanju’. I. *C. sinensis* ‘Liu Cheng’. J. *C. reticulata Blanco* ‘Shatangju’. K. *C. sinensis* ‘Gailiang Cheng’. L. *C. reticulata Blanco* ‘Wokan’. M. *C. reticulata Blanco* ‘Kinokuni’. N. *C. reticulata Blanco* ‘Gongkan’. O. Healthy citrus ‘Shatangju’ fruit with color changing started from flower end. P. HLB-affected ‘Shatangju’ fruit with color changing started from stem end. Q. Healthy ‘Shatangju’ fruit. R. HLB-affected ‘Shatangju’ fruit. The red arrow indicated the different color change in the vascular columella under the peduncle between diseased and healthy fruit.

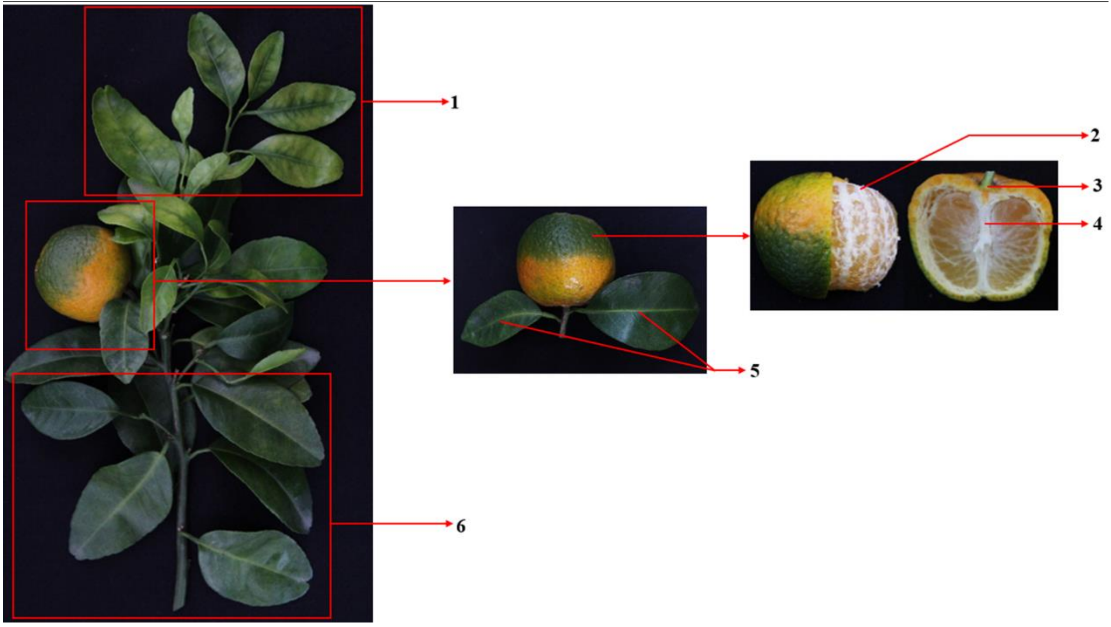

Fig. S3. Sampling diagram of Huanglongbing-affected citrus branch with fruit (*Citrus reticulata Blanco* ‘Shatangju’ as example). 1. Leaves of new flush; 2. Fruit pith; 3. Peduncle; 4. Central axis; 5. Leaves that adjacent to fruit; 6. Mature leaves or stem bark.

Table S1. Differentially expressed genes of "*Candidatus* Liberibacter asiaticus" between leaf midribs HiSeq data and fruit pith HiSeq data obtained by GFOLD V1.1.4. *Gene symbol referenced to genome of CLas A4 strain (CP010804.2)

No.	Gene symbol*	Log2 Fold change	COG function description
1	CD16_RS05215	-4.56881	Not Classifies in COG function categories
2	CD16_RS04485	-3.39995	Not Classifies in COG function categories
3	CD16_RS04950	-3.38824	Cell wall formation
4	CD16_RS04330	-3.15378	Part of the phosphoribosylformylglycinamidine synthase complex
5	CD16_RS00670	-3.08339	Involved in the binding of tRNA to the ribosomes
6	CD16_RS01170	-3.01627	Belongs to the bacterial ribosomal protein bL32 family
7	CD16_RS04635	-2.98385	COG0720 6-pyruvoyl-tetrahydropterin synthase
8	CD16_RS00455	-2.88076	Phosphorylation of dTMP to form dTDP in both de novo and salvage pathways of dTTP synthesis
9	CD16_RS00440	-2.83185	TatD family
10	CD16_RS00540	-2.83185	Ribosomal protein L17
11	CD16_RS02835	-2.73874	3'-5' exonuclease
12	CD16_RS05100	-2.70607	Not Classifies in COG function categories
13	CD16_RS02650	-2.63021	Not Classifies in COG function categories
14	CD16_RS00475	-2.60358	Permease
15	CD16_RS03155	-2.56881	Catalyzes the formation of phosphatidylethanolamine (PtdEtn) from phosphatidylserine (PtdSer)
16	CD16_RS03135	-2.52151	COG0500 SAM-dependent methyltransferases
17	CD16_RS04480	-2.50992	Belongs to the class I-like SAM-binding methyltransferase superfamily. RNA M5U methyltransferase family
18	CD16_RS04030	-2.4823	Not Classifies in COG function categories
19	CD16_RS04450	-2.4823	double-stranded DNA 3'-5' exodeoxyribonuclease activity
20	CD16_RS02050	-2.45334	Endonuclease that specifically degrades the RNA of RNA- DNA hybrids
21	CD16_RS02265	-2.45334	Zn-dependent hydrolases, including glyoxylases

22	CD16_RS04960	-2.42586	Cell wall formation
23	CD16_RS02310	-2.41681	This protein specifically catalyzes the removal of signal peptides from prolipoproteins
24	CD16_RS01560	-2.39889	Catalyzes the complicated ring closure reaction between the two acyclic compounds
25	CD16_RS00360	-2.36392	P-P-bond-hydrolysis-driven protein transmembrane transporter activity
26	CD16_RS04155	-2.33065	NYN domain
27	CD16_RS02220	-2.31972	DNA polymerase III is a complex, multichain enzyme responsible for most of the replicative synthesis in bacteria.
28	CD16_RS03325	-2.21518	Protein conserved in bacteria
29	CD16_RS01995	-2.19958	Specifically methylates the N7 position of guanine in position 527 of 16S rRNA
30	CD16_RS02165	-2.16672	Zinc metalloprotease
31	CD16_RS01010	-2.15378	Binds together with S18 to 16S ribosomal RNA
32	CD16_RS05315	-2.15378	Catalyzes the hydrolysis of UDP-3-O-myristoyl-N- acetylglucosamine to form UDP-3-O-myristoylglucosamine and acetate
33	CD16_RS03425	-2.15283	flagellar
34	CD16_RS05095	-2.13598	Not Classifies in COG function categories
35	CD16_RS01040	-2.06631	Toxin biosynthetic process
36	CD16_RS01985	-2.06631	Exhibits a very high intrinsic GTPase hydrolysis rate
37	CD16_RS04905	-2.02449	ATP synthesis coupled proton transport
38	CD16_RS01180	-2.01627	Flavodoxin reductases (ferredoxin-NADPH reductases) family 1
39	CD16_RS04755	-1.98385	Not Classifies in COG function categories
40	CD16_RS03675	-1.95738	Not Classifies in COG function categories
41	CD16_RS01590	-1.94432	Repressor
42	CD16_RS01800	-1.93655	Required for the insertion and or proper folding and or complex formation of integral membrane proteins into the membrane.
43	CD16_RS04750	-1.93138	COG3772 Phage-related lysozyme (muraminidase)
44	CD16_RS03080	-1.92112	Catalyzes the dephosphorylation of undecaprenyl diphosphate (UPP). Confers resistance to bacitracin
45	CD16_RS01075	-1.8561	Glycine betaine transport system, permease

46	CD16_RS00745	-1.85121	Catalyzes the condensation of (S)-aspartate-beta- semialdehyde (S)-ASA and pyruvate to 4-hydroxy-tetrahydrodipicolinate (HTPA)
47	CD16_RS00435	-1.85	Metal-dependent hydrolases of the beta-lactamase superfamily I
48	CD16_RS00610	-1.80546	One of the proteins that surrounds the polypeptide exit tunnel on the outside of the subunit
49	CD16_RS02480	-1.80546	An accessory protein needed during the final step in the assembly of 30S ribosomal subunit
50	CD16_RS01625	-1.78625	Belongs to the BI1 family
51	CD16_RS05120	-1.77526	Not Classifies in COG function categories
52	CD16_RS01400	-1.76146	Belongs to the pseudouridine synthase RsuA family
53	CD16_RS02320	-1.76146	Periplasmic serine proteases (ClpP class)
54	CD16_RS04425	-1.76146	Transferase activity, transferring acyl groups
55	CD16_RS00510	-1.73026	Integral membrane protein
56	CD16_RS04310	-1.73026	Belongs to the glutaredoxin family. Monothiol subfamily
57	CD16_RS01930	-1.72751	Component of the acetyl coenzyme A carboxylase (ACC) complex
58	CD16_RS02545	-1.72751	PhoQ Sensor
59	CD16_RS02465	-1.69434	Catalyzes the oxidative decarboxylation of 6- phosphogluconate to ribulose 5-phosphate and CO(2)
60	CD16_RS04230	-1.69434	COG0210 Superfamily I DNA and RNA helicases
61	CD16_RS04390	-1.66835	Efflux protein
62	CD16_RS00210	-1.66192	ABC-type amino acid transport signal transduction systems, periplasmic component domain
63	CD16_RS01235	-1.6588	Assembles around the rod to form the L-ring and probably protects the motor basal body from shearing forces during rotation
64	CD16_RS03560	-1.6588	Co Zn Cd cation transporter, cation efflux family
65	CD16_RS02675	-1.64598	The pyruvate dehydrogenase complex catalyzes the overall conversion of pyruvate to acetyl-CoA and CO(2)
66	CD16_RS04565	-1.61772	COG3407 Mevalonate pyrophosphate decarboxylase
67	CD16_RS03820	-1.61462	Belongs to the aspartokinase family
68	CD16_RS04555	-1.59071	Not Classifies in COG function categories
69	CD16_RS00570	-1.56881	Binds to the 23S rRNA

70	CD16_RS01160	-1.56881	Catalyzes the conversion of D-ribulose 5-phosphate to formate and 3,4-dihydroxy-2-butanone 4-phosphate
71	CD16_RS02710	-1.56881	Reversible hydration of carbon dioxide
72	CD16_RS02945	-1.56881	Produces ATP from ADP in the presence of a proton gradient across the membrane
73	CD16_RS04250	-1.56881	SsrA-binding protein SmpB
74	CD16_RS00895	-1.54891	Lytic murein transglycosylase
75	CD16_RS03800	-1.52929	Catalyzes the attachment of proline to tRNA(Pro) in a two-step reaction
76	CD16_RS01250	-1.52568	Involved in the assembly process of the P-ring formation
77	CD16_RS01200	-1.52442	Ribonuclease
78	CD16_RS03085	-1.52151	COG0457 FOG TPR repeat
79	CD16_RS00595	-1.49842	One of the primary rRNA binding proteins
80	CD16_RS04585	-1.47195	Involved in the biosynthesis of isoprenoids
81	CD16_RS03880	-1.46615	protein tyrosine kinase activity
82	CD16_RS04115	-1.46615	Not Classifies in COG function categories
83	CD16_RS05530	-1.46615	Not Classifies in COG function categories
84	CD16_RS01945	-1.46346	One of the proteins required for the normal export of preproteins out of the cell cytoplasm
85	CD16_RS05565	-1.46346	Not Classifies in COG function categories
86	CD16_RS00755	-1.45334	Belongs to the phosphoglycerate mutase family. BPG- dependent PGAM subfamily
87	CD16_RS03195	-1.45334	Phosphoribosylformylglycinamidine cyclo-ligase
88	CD16_RS02090	-1.45234	Participates in both transcription termination and antitermination
89	CD16_RS00785	-1.43496	Sporulation related domain
90	CD16_RS04140	-1.423	amino acids such as threonine
91	CD16_RS03955	-1.41681	Involved in protein export
92	CD16_RS00105	-1.41504	This protein promotes the GTP-dependent binding of aminoacyl-tRNA to the A-site of ribosomes during protein biosynthesis
93	CD16_RS01785	-1.39889	2-deoxycytidine 5-triphosphate deaminase
94	CD16_RS05425	-1.38902	Outer membrane

95	CD16_RS00980	-1.38661	Provides the rickettsial cell with host ATP in exchange for rickettsial ADP
96	CD16_RS04600	-1.36928	Not Classifies in COG function categories
97	CD16_RS02730	-1.34653	transcriptional regulatory protein
98	CD16_RS03180	-1.34653	TadE-like protein
99	CD16_RS00370	-1.34642	Not Classifies in COG function categories
100	CD16_RS01455	-1.34642	Global regulator CarD
101	CD16_RS04300	-1.34642	Not Classifies in COG function categories
102	CD16_RS00915	-1.33502	Peroxiredoxin
103	CD16_RS03110	-1.32089	Recombinase XerD
104	CD16_RS00120	-1.31247	Belongs to the D-isomer specific 2-hydroxyacid dehydrogenase family
105	CD16_RS01885	-1.31247	Protease subunit of a proteasome-like degradation complex believed to be a general protein degrading machinery
106	CD16_RS03765	-1.29143	Belongs to the complex I subunit 6 family
107	CD16_RS04765	-1.29143	COG0396 ABC-type transport system involved in Fe-S cluster assembly, ATPase component
108	CD16_RS03055	-1.2875	Has lipid A 3-O-deacylase activity
109	CD16_RS00940	-1.28513	Forms passive diffusion pores that allow small molecular weight hydrophilic materials across the outer membrane
110	CD16_RS02450	-1.27113	ABC-type Mn2 Zn2 transport systems, permease components
111	CD16_RS00325	-1.26842	Catalyzes the attachment of alanine to tRNA(Ala) in a two-step reaction
112	CD16_RS02435	-1.26069	Pilus assembly protein
113	CD16_RS01575	-1.24689	Lipopolysaccharide core biosynthesis mannosyltransferase
114	CD16_RS02170	-1.24689	Part of the outer membrane protein assembly complex
115	CD16_RS02285	-1.24689	Involved in the regulation of the intracellular balance of NAD and NADP, and is a key enzyme in the biosynthesis of NADP
116	CD16_RS03925	-1.24689	Histidine phosphotransferase C-terminal domain
117	CD16_RS04225	-1.23837	DUF218 domain
118	CD16_RS02570	-1.22304	Belongs to the prokaryotic GSH synthase family
119	CD16_RS00280	-1.20016	A type II topoisomerase

120	CD16_RS01910	-1.19958	double-strand break repair protein AddB
121	CD16_RS04775	-1.19958	Belongs to the class-V pyridoxal-phosphate-dependent aminotransferase family
122	CD16_RS04720	-1.1903	Succinyl-CoA synthetase functions in the citric acid cycle (TCA)
123	CD16_RS01445	-1.14255	Membrane protein TerC, possibly involved in tellurium resistance
124	CD16_RS04605	-1.13883	electron transfer flavoprotein-ubiquinone oxidoreductase
125	CD16_RS04810	-1.13585	Not Classifies in COG function categories
126	CD16_RS05320	-1.13388	Essential cell division protein that forms a contractile ring structure (Z ring) at the future cell division site
127	CD16_RS02700	-1.12698	Belongs to the class I-like SAM-binding methyltransferase superfamily. RsmB NOP family
128	CD16_RS03415	-1.12403	flagellar hook-associated protein
129	CD16_RS01410	-1.11815	Involved in the biosynthesis of the central metabolite phospho-alpha-D-ribosyl-1-pyrophosphate (PRPP)
130	CD16_RS01580	-1.10938	Belongs to the pyruvate kinase family
131	CD16_RS03400	-1.10938	Flagellar hook-length control protein FliK
132	CD16_RS03730	-1.10938	NDH-1 shuttles electrons from NADH, via FMN and iron- sulfur (Fe-S) centers, to quinones in the respiratory chain
133	CD16_RS01565	-1.10602	Belongs to the CarB family
134	CD16_RS01050	-1.08996	Belongs to the short-chain dehydrogenases reductases (SDR) family
135	CD16_RS01635	-1.08769	Confers DNA tethering and processivity to DNA polymerases and other proteins. Acts as a clamp, forming a ring around DNA
136	CD16_RS02110	-1.08276	Involved in mRNA degradation
137	CD16_RS00695	-1.06631	biotin carboxylase
138	CD16_RS03620	-1.06631	DNA polymerase III alpha subunit
139	CD16_RS03305	-1.05424	Ligates lysine onto the cytidine present at position 34 of the AUA codon-specific tRNA(Ile)
140	CD16_RS02055	-1.05224	Belongs to the pseudomonas-type ThrB family
141	CD16_RS00215	-1.04525	ABC-type amino acid transport system, permease component
142	CD16_RS04675	-1.0437	EVE domain
143	CD16_RS00950	-1.0383	Belongs to the class-II aminoacyl-tRNA synthetase family. Phe-tRNA synthetase alpha subunit type 1 subfamily
144	CD16_RS00870	-1.02449	dihydroorotase

145	CD16_RS03070	-1.02449	COG0451 Nucleoside-diphosphate-sugar epimerases
146	CD16_RS03160	-1.02449	ABC-type transport system involved in Fe-S cluster assembly, permease and ATPase components
147	CD16_RS04985	-1.02449	Catalyzes the addition of meso-diaminopimelic acid to the nucleotide precursor UMAG in the biosynthesis of bacterial cell-wall peptidoglycan
148	CD16_RS04880	1.01116	Not Classifies in COG function categories
149	CD16_RS04890	1.05695	Not Classifies in COG function categories
150	CD16_RS04875	1.12774	Not Classifies in COG function categories
151	CD16_RS04885	2.19513	Not Classifies in COG function categories

*Gene symbol and gene name referenced to CLas A4 strain (CP010804.2)

Table S2. Top 100 up-regulated DEGs in CLas-infected leaf midribs compared with CLas-infected fruit pith.

No.	Gene_id	Read count (leaf midribs)	Read count (fruit pith)	Log2 Fold change.	pvalue	qvalue	Blast swiss prot
1	Ciclev10022041m.v1.0	1224.00	0.00	11.26	1.03E-231	2.54E-229	sp|P13087|MIRA_RICDU Miraculin OS=Richadella dulcifica PE=1 SV=3//7.55454e-14
2	Ciclev10006105m.v1.0	1140.22	1.03	10.11	4.22E-265	1.26E-262	sp|O50001|PRU1_PRUAR Major allergen Pru ar 1 OS=Prunus armeniaca PE=1 SV=1//3.25881e-41
3	Ciclev10022909m.v1.0	1753.15	2.57	9.41	0	0	sp|P19873|ITH5_CUCMA Inhibitor of trypsin and hageman factor OS=Cucurbita maxima PE=1 SV=1//1.90016e-14
4	Ciclev10013661m.v1.0	225.42	0.00	8.77	1.58E-66	7.87E-65	Not found
5	Ciclev10008091m.v1.0	217.58	0.00	8.72	9.39E-65	4.38E-63	sp|P93338|GAPN_NICPL NADP-dependent glyceraldehyde-3-phosphate dehydrogenase OS=Nicotiana plumbaginifolia GN=GAPN PE=2 SV=1//0
6	Ciclev10031765m.v1.0	196.97	0.00	8.62	1.19E-59	5.01E-58	sp|P46283|S17P_ARATH Sedoheptulose-1,7-bisphosphatase, chloroplastic OS=Arabidopsis thaliana GN=At3g55800 PE=1 SV=1//0
7	Ciclev10005136m.v1.0	99.53	0.00	8.59	4.05E-31	6.69E-30	sp|Q66283|CP_CSVMV Putative Polyprotein CP OS=Cassava vein mosaic virus GN=ORF 1 PE=4 SV=1//1.17136e-09
8	Ciclev10022001m.v1.0	485.63	1.29	8.56	1.41E-145	1.94E-143	sp|P13087|MIRA_RICDU Miraculin OS=Richadella dulcifica PE=1 SV=3//6.99519e-25
9	Ciclev10022906m.v1.0	308.00	1.03	8.22	1.78E-97	1.46E-95	sp|P19873|ITH5_CUCMA Inhibitor of trypsin and hageman factor OS=Cucurbita maxima PE=1 SV=1//2.81673e-15
10	Ciclev10024853m.v1.0	152.62	0.00	8.21	2.57E-49	8.16E-48	sp|O65924|Y2921_ARATH Putative leucine-rich repeat receptor-like protein kinase At2g19210 OS=Arabidopsis thaliana GN=At2g19210 PE=2 SV=1//0

11	Ciclev10012964m.v1.0	420.37	1.54	8.09	8.97E-135	1.14E-132	Not found
12	Ciclev10004731m.v1.0	134.25	0.00	8.07	2.05E-44	5.51E-43	sp|P55229|GLGL1_ARATH Glucose-1-phosphate adenylyltransferase large subunit 1, chloroplastic OS=Arabidopsis thaliana GN=ADG2 PE=1 SV=3//0
13	Ciclev10002099m.v1.0	503.10	2.06	7.93	3.09E-164	5.09E-162	sp|Q02060|PSBS_SPIOL Photosystem II 22 kDa protein, chloroplastic OS=Spinacia oleracea GN=PSBS PE=1 SV=1//8.87197e-99
14	Ciclev10002650m.v1.0	62.20	0.00	7.92	7.06E-22	7.16E-21	Not found
15	Ciclev10001677m.v1.0	181.66	0.00	7.88	6.01E-61	2.63E-59	sp|D4N501|DIOX2_PAPSO Probable 2-oxoglutarate/Fe(II)-dependent dioxygenase OS=Papaver somniferum GN=DIOX2 PE=2 SV=1//3.1161e-67
16	Ciclev10031639m.v1.0	797.58	3.60	7.79	5.90E-264	1.71E-261	sp|Q6XBF8|CDR1_ARATH Aspartic proteinase CDR1 OS=Arabidopsis thaliana GN=CDR1 PE=1 SV=1//8.18259e-135
17	Ciclev10022706m.v1.0	1575.00	7.21	7.77	0	0	Not found
18	Ciclev10012995m.v1.0	48.16	0.00	7.55	4.76E-18	3.79E-17	sp|Q67A25|NCS_THLFG S-norcoclaurine synthase OS=Thalictrum flavum subsp. glaucum PE=1 SV=1//3.65698e-14
19	Ciclev10031241m.v1.0	328.16	1.80	7.51	1.19E-113	1.16E-111	sp|P95245|PHLC_MYCTU Phospholipase C 3 OS=Mycobacterium tuberculosis GN=plcC PE=3 SV=2//5.26606e-22
20	Ciclev10024449m.v1.0	89.00	0.00	7.48	2.98E-32	5.22E-31	sp|O23547|EXLB1_ARATH Expansin-like B1 OS=Arabidopsis thaliana GN=EXLB1 PE=2 SV=2//1.26253e-97
21	Ciclev10021095m.v1.0	90.72	0.00	7.46	6.74E-33	1.20E-31	sp|Q94LX1|CLH1_CITUN Chlorophyllase-1, chloroplastic OS=Citrus unshiu PE=2 SV=1//2.91505e-117
22	Ciclev10021442m.v1.0	86.31	0.00	7.43	1.74E-31	2.91E-30	sp|Q9SG92|MES17_ARATH Methylesterase 17 OS=Arabidopsis thaliana GN=MES17 PE=1 SV=1//4.50087e-66
23	Ciclev10020613m.v1.0	81.76	0.00	7.35	3.55E-30	5.68E-29	sp|Q9STX2|VEP1_ARATH 3-oxo-Delta(4,5)-steroid 5-beta-reductase OS=Arabidopsis thaliana GN=VEP1 PE=1 SV=1//2.71214e-96
24	Ciclev10006614m.v1.0	41.89	0.00	7.35	3.00E-16	2.08E-15	sp|Q43077|AMO_PEA Primary amine oxidase OS=Pisum sativum PE=1 SV=1//9.54426e-27

25	Ciclev10006188m.v1.0	79.00	0.00	7.30	2.26E-29	3.46E-28	Not found
26	Ciclev10009650m.v1.0	77.80	0.00	7.28	5.05E-29	7.66E-28	sp|P59082|LFS_ALLCE Lachrymatory-factor synthase OS=Allium cepa GN=LFS PE=1 SV=1//1.13321e-15
27	Ciclev10000991m.v1.0	320.39	2.06	7.28	5.58E-114	5.57E-112	sp|Q39134|AAP3_ARATH Amino acid permease 3 OS=Arabidopsis thaliana GN=AAP3 PE=1 SV=2//0
28	Ciclev10018615m.v1.0	76.68	0.00	7.26	1.08E-28	1.60E-27	Not found
29	Ciclev10023773m.v1.0	74.22	0.00	7.21	5.75E-28	8.11E-27	sp|Q93ZR6|WSD1_ARATH O-acyltransferase WSD1 OS=Arabidopsis thaliana GN=WSD1 PE=2 SV=1//2.07424e-76
30	Ciclev10029583m.v1.0	37.71	0.00	7.19	5.17E-15	3.23E-14	Not found
31	Ciclev10011511m.v1.0	112.52	0.00	7.19	1.20E-41	3.01E-40	sp|P37122|C76A2_SOLME Cytochrome P450 76A2 OS=Solanum melongena GN=CYP76A2 PE=2 SV=1//1.51582e-167
32	Ciclev10009700m.v1.0	37.48	0.00	7.19	6.03E-15	3.73E-14	sp|Q9LTF7|MYB82_ARATH Transcription factor MYB82 OS=Arabidopsis thaliana GN=MYB82 PE=1 SV=1//8.68683e-20
33	Ciclev10033071m.v1.0	222.58	1.54	7.17	9.34E-81	5.99E-79	sp|Q9XIF4|TRXH7_ARATH Thioredoxin H7 OS=Arabidopsis thaliana GN=TRX7 PE=2 SV=1//7.71367e-34
34	Ciclev10002084m.v1.0	74.07	0.00	7.17	4.77E-28	6.77E-27	sp|Q84WU2|UBP13_ARATH Ubiquitin carboxyl-terminal hydrolase 13 OS=Arabidopsis thaliana GN=UBP13 PE=1 SV=1//1.06889e-09
35	Ciclev10003554m.v1.0	73.92	0.00	7.17	5.28E-28	7.45E-27	Not found
36	Ciclev10014538m.v1.0	147.84	1.03	7.17	2.74E-54	1.02E-52	sp|Q9SXS2|SUT33_ARATH Probable sulfate transporter 3.3 OS=Arabidopsis thaliana GN=SULTR3;3 PE=2 SV=2//0
37	Ciclev10021884m.v1.0	143.58	1.03	7.12	5.11E-53	1.84E-51	sp|O23547|EXLB1_ARATH Expansin-like B1 OS=Arabidopsis thaliana GN=EXLB1 PE=2 SV=2//2.66142e-97
38	Ciclev10011447m.v1.0	35.17	0.00	7.09	3.02E-14	1.76E-13	sp|Q38931|FKB62_ARATH Peptidyl-prolyl cis-trans isomerase FKBP62 OS=Arabidopsis thaliana GN=FKBP62 PE=1 SV=2//0
39	Ciclev10016980m.v1.0	1815.28	13.65	7.06	0	0	Not found

40	Ciclev10014858m.v1.0	63.62	0.00	6.99	9.11E-25	1.11E-23	sp|Q9SBQ9|F3PH_PETHY Flavonoid 3'-monooxygenase OS=Petunia hybrida GN=CYP75B2 PE=2 SV=1//1.67059e-93
41	Ciclev10014199m.v1.0	4199.80	33.47	6.97	0	0	sp|O24370|LOX21_SOLTU Linoleate 13S-lipoxygenase 2-1, chloroplastic OS=Solanum tuberosum GN=LOX2.1 PE=1 SV=1//0
42	Ciclev10009184m.v1.0	62.20	0.00	6.96	2.49E-24	2.94E-23	sp|Q9FG33|LRKS5_ARATH Probable L-type lectin-domain containing receptor kinase S.5 OS=Arabidopsis thaliana GN=LECRKS5 PE=2 SV=1//2.14957e-18
43	Ciclev10031329m.v1.0	127.31	1.03	6.95	4.49E-48	1.34E-46	sp|Q9XF43|KCS6_ARATH 3-ketoacyl-CoA synthase 6 OS=Arabidopsis thaliana GN=CUT1 PE=1 SV=1//0
44	Ciclev10019837m.v1.0	63.24	0.00	6.94	9.33E-25	1.14E-23	sp|Q9ZU96|Y2168_ARATH Ankyrin repeat-containing protein At2g01680 OS=Arabidopsis thaliana GN=At2g01680 PE=1 SV=1//5.86224e-14
45	Ciclev10013191m.v1.0	366.46	3.09	6.89	1.02E-135	1.34E-133	sp|Q43681|NLTP_VIGUN Probable non-specific lipid-transfer protein AKCS9 OS=Vigna unguiculata PE=2 SV=1//2.02045e-24
46	Ciclev10000810m.v1.0	28.97	0.00	6.81	2.58E-12	1.24E-11	sp|A6P6V9|CBDAS_CANSA Cannabidiolic acid synthase OS=Cannabis sativa GN=CBDAS PE=1 SV=1//4.35832e-154
47	Ciclev10006217m.v1.0	54.80	0.00	6.78	5.18E-22	5.31E-21	sp|O64645|SOC1_ARATH MADS-box protein SOC1 OS=Arabidopsis thaliana GN=SOC1 PE=1 SV=1//5.83075e-17
48	Ciclev10005245m.v1.0	27.70	0.00	6.75	6.60E-12	3.03E-11	sp|P94111|STS1_ARATH Strictosidine synthase 1 OS=Arabidopsis thaliana GN=SS1 PE=2 SV=2//1.19822e-60
49	Ciclev10007738m.v1.0	55.03	0.00	6.74	3.59E-22	3.73E-21	sp|Q8H1Z0|CER3_ARATH Protein ECERIFERUM 3 OS=Arabidopsis thaliana GN=CER3 PE=1 SV=1//0
50	Ciclev10012033m.v1.0	51.07	0.00	6.67	8.15E-21	7.73E-20	sp|B6VJS4|ROMT_VITVI Trans-resveratrol di-O-methyltransferase OS=Vitis vinifera GN=ROMT PE=1 SV=2//9.21634e-163
51	Ciclev10015126m.v1.0	50.77	0.00	6.67	1.02E-20	9.54E-20	sp|Q93ZR6|WSD1_ARATH O-acyltransferase WSD1 OS=Arabidopsis thaliana GN=WSD1 PE=2 SV=1//6.91839e-42
52	Ciclev10028831m.v1.0	156.72	1.54	6.66	1.90E-60	8.17E-59	sp|P06215|CHIT_PHAVU Endochitinase OS=Phaseolus vulgaris PE=1 SV=1//4.09785e-111

53	Ciclev10014991m.v1.0	258.87	2.57	6.65	9.56E-99	8.10E-97	sp|P48417|CP74_LINUS Allene oxide synthase, chloroplastic OS=Linum usitatissimum GN=CYP74A PE=1 SV=1//1.04097e-124
54	Ciclev10026869m.v1.0	51.00	0.00	6.63	7.18E-21	6.83E-20	Not found
55	Ciclev10022063m.v1.0	101.47	1.03	6.62	6.42E-40	1.54E-38	sp|O04138|CHI4_ORYSJ Chitinase 4 OS=Oryza sativa subsp. japonica GN=Cht4 PE=2 SV=2//3.40713e-91
56	Ciclev10028277m.v1.0	50.40	0.00	6.61	1.12E-20	1.05E-19	sp|Q8VWZ7|C76B6_CATRO Geraniol 8-hydroxylase OS=Catharanthus roseus GN=CYP76B6 PE=1 SV=1//0
57	Ciclev10009194m.v1.0	24.64	0.00	6.58	6.61E-11	2.74E-10	sp|Q9FG33|LRKS5_ARATH Probable L-type lectin-domain containing receptor kinase S.5 OS=Arabidopsis thaliana GN=LECRKS5 PE=2 SV=1//3.95748e-13
58	Ciclev10014792m.v1.0	24.27	0.00	6.56	8.79E-11	3.61E-10	sp|Q9C9H7|RLP12_ARATH Receptor-like protein 12 OS=Arabidopsis thaliana GN=RLP12 PE=2 SV=2//2.08418e-22
59	Ciclev10022211m.v1.0	48.08	0.00	6.55	6.45E-20	5.74E-19	sp|P32765|ASP_THECC 21 kDa seed protein OS=Theobroma cacao GN=ASP PE=2 SV=1//1.58015e-31
60	Ciclev10027467m.v1.0	47.71	0.00	6.53	8.57E-20	7.55E-19	sp|O64793|Y1675_ARATH G-type lectin S-receptor-like serine/threonine-protein kinase At1g67520 OS=Arabidopsis thaliana GN=At1g67520 PE=2 SV=3//2.07111e-43
61	Ciclev10000924m.v1.0	46.29	0.00	6.53	2.97E-19	2.53E-18	sp|O65782|C83B1_ARATH Cytochrome P450 83B1 OS=Arabidopsis thaliana GN=CYP83B1 PE=1 SV=1//1.51414e-174
62	Ciclev10006226m.v1.0	47.34	0.00	6.52	1.14E-19	9.96E-19	sp|Q8RWI9|AB15G_ARATH ABC transporter G family member 15 OS=Arabidopsis thaliana GN=ABCG15 PE=2 SV=2//1.34961e-20
63	Ciclev10029614m.v1.0	23.45	0.00	6.51	1.65E-10	6.58E-10	sp|Q9FK81|Y5258_ARATH Uncharacterized protein At5g22580 OS=Arabidopsis thaliana GN=At5g22580 PE=1 SV=1//1.02127e-38
64	Ciclev10014671m.v1.0	23.37	0.00	6.50	1.75E-10	6.94E-10	Not found
65	Ciclev10025330m.v1.0	268.20	3.09	6.44	2.98E-104	2.66E-102	sp|P0C897|Y3264_ARATH Putative UPF0481 protein At3g02645 OS=Arabidopsis thaliana GN=At3g02645 PE=3 SV=1//5.0199e-130
66	Ciclev10013224m.v1.0	41.59	0.00	6.38	1.11E-17	8.60E-17	Not found

67	Ciclev10015234m.v1.0	143.36	1.80	6.31	3.29E-57	1.32E-55	sp|Q8GT20|BEBT_TOBAC Benzyl alcohol O-benzoyltransferase OS=Nicotiana tabacum GN=HSR201 PE=1 SV=1//1.24601e-164
68	Ciclev10019727m.v1.0	716.57	9.01	6.31	3.20E-278	9.95E-276	sp|Q9LSF8|C82G1_ARATH Cytochrome P450 82G1 OS=Arabidopsis thaliana GN=CYP82G1 PE=1 SV=1//8.56572e-176
69	Ciclev10009128m.v1.0	58.76	0.00	6.25	1.40E-24	1.68E-23	sp|Q8S9H7|DIV_ANTMA Transcription factor DIVARICATA OS=Antirrhinum majus GN=DIVARICATA PE=2 SV=1//6.536e-26
70	Ciclev10020850m.v1.0	38.00	0.00	6.25	1.86E-16	1.31E-15	Not found
71	Ciclev10031848m.v1.0	38.60	0.00	6.23	1.02E-16	7.38E-16	sp|P52839|SOT12_ARATH Cytosolic sulfotransferase 12 OS=Arabidopsis thaliana GN=SOT12 PE=1 SV=2//9.88807e-115
72	Ciclev10032182m.v1.0	37.93	0.00	6.20	1.75E-16	1.24E-15	sp|Q9ZVJ6|ANXD4_ARATH Annexin D4 OS=Arabidopsis thaliana GN=ANN4 PE=2 SV=1//2.43614e-107
73	Ciclev10013188m.v1.0	188.08	2.57	6.19	3.08E-75	1.80E-73	sp|P82353|NLTP2_PRUAR Non-specific lipid-transfer protein 2 OS=Prunus armeniaca PE=1 SV=1//8.00341e-25
74	Ciclev10028195m.v1.0	1987.98	27.29	6.19	0	0	sp|Q00081|GLGL1_SOLTU Glucose-1-phosphate adenylyltransferase large subunit 1 (Fragment) OS=Solanum tuberosum GN=AGPS1 PE=2 SV=1//0
75	Ciclev10011671m.v1.0	18.74	0.00	6.19	6.84E-09	2.25E-08	sp|Q9SD53|Y3720_ARATH UPF0481 protein At3g47200 OS=Arabidopsis thaliana GN=At3g47200 PE=1 SV=1//3.73982e-38
76	Ciclev10024410m.v1.0	37.48	0.00	6.19	2.51E-16	1.75E-15	sp|O23547|EXLB1_ARATH Expansin-like B1 OS=Arabidopsis thaliana GN=EXLB1 PE=2 SV=2//9.19265e-86
77	Ciclev10011096m.v1.0	37.03	0.00	6.17	3.59E-16	2.48E-15	sp|Q9M353|CHX20_ARATH Cation/H(+) antiporter 20 OS=Arabidopsis thaliana GN=CHX20 PE=2 SV=1//0
78	Ciclev10028242m.v1.0	55.48	0.00	6.17	1.91E-23	2.16E-22	sp|Q8W3M4|Y4744_ARATH Uncharacterized protein At4g06744 OS=Arabidopsis thaliana GN=At4g06744 PE=2 SV=1//2.24581e-110
79	Ciclev10022574m.v1.0	18.44	0.00	6.16	8.72E-09	2.82E-08	sp|P16273|PRPX_HORVU Pathogen-related protein OS=Hordeum vulgare PE=2 SV=2//7.21776e-68

80	Ciclev10025893m.v1.0	35.69	0.00	6.16	1.19E-15	7.78E-15	sp|Q9FLN0|GLIP1_ARATH GDSL esterase/lipase 1 OS=Arabidopsis thaliana GN=GLIP1 PE=1 SV=1//1.17999e-128
81	Ciclev10000235m.v1.0	310.76	4.38	6.15	1.83E-123	2.05E-121	sp|Q3KTM0|FRO7_ARATH Ferric reduction oxidase 7, chloroplastic OS=Arabidopsis thaliana GN=FRO7 PE=2 SV=1//1.01414e-116
82	Ciclev10002547m.v1.0	35.17	0.00	6.14	1.81E-15	1.17E-14	Not found
83	Ciclev10018045m.v1.0	36.14	0.00	6.13	7.39E-16	4.95E-15	sp|Q84LB2|AFS1_MALDO (E,E)-alpha-farnesene synthase OS=Malus domestica GN=AFS1 PE=1 SV=2//3.06147e-108
84	Ciclev10009617m.v1.0	71.98	1.03	6.13	5.06E-30	7.98E-29	Not found
85	Ciclev10033930m.v1.0	35.84	0.00	6.12	9.40E-16	6.24E-15	sp|Q8W3Z1|BAMS_BETPL Beta-amyrin synthase OS=Betula platyphylla GN=OSCBPY PE=1 SV=1//0
86	Ciclev10014930m.v1.0	35.69	0.00	6.12	1.06E-15	6.99E-15	sp|Q9FMY1|C86B1_ARATH Cytochrome P450 86B1 OS=Arabidopsis thaliana GN=CYP86B1 PE=2 SV=1//1.38384e-110
87	Ciclev10020150m.v1.0	17.85	0.00	6.12	1.42E-08	4.48E-08	sp|Q60DN5|PROT1_ORYSJ Proline transporter 1 OS=Oryza sativa subsp. japonica GN=PROT1 PE=2 SV=1//8.23423e-110
88	Ciclev10002362m.v1.0	70.78	1.03	6.10	1.32E-29	2.04E-28	sp|P42820|CHIP_BETVU Acidic endochitinase SP2 OS=Beta vulgaris GN=SP2 PE=1 SV=1//5.18762e-72
89	Ciclev10018908m.v1.0	17.62	0.00	6.10	1.71E-08	5.32E-08	sp|C0LGN2|Y3148_ARATH Probable leucine-rich repeat receptor-like serine/threonine-protein kinase At3g14840 OS=Arabidopsis thaliana GN=LRR-RLK PE=1 SV=1//0
90	Ciclev10009284m.v1.0	33.75	0.00	6.08	5.71E-15	3.54E-14	sp|Q12288|YL126_YEAST Putative glutamine amidotransferase YLR126C OS=Saccharomyces cerevisiae (strain ATCC 204508 / S288c) GN=YLR126C PE=1 SV=1//4.02841e-19
91	Ciclev10025646m.v1.0	34.42	0.00	6.06	2.98E-15	1.88E-14	sp|Q9FMF5|RPT3_ARATH Root phototropism protein 3 OS=Arabidopsis thaliana GN=RPT3 PE=1 SV=2//1.61583e-74
92	Ciclev10022911m.v1.0	33.30	0.00	6.06	8.23E-15	5.03E-14	sp|P32765|ASP_THECC 21 kDa seed protein OS=Theobroma cacao GN=ASP PE=2 SV=1//6.28212e-32

93	Ciclev10022156m.v1.0	33.00	0.00	6.04	1.05E-14	6.35E-14	sp|P32765|ASP_THECC 21 kDa seed protein OS=Theobroma cacao GN=ASP PE=2 SV=1//3.32967e-26
94	Ciclev10012464m.v1.0	32.70	0.00	6.03	1.34E-14	8.05E-14	sp|P36591|DYR_SCHPO Dihydrofolate reductase OS=Schizosaccharomyces pombe (strain 972 / ATCC 24843) GN=dfr1 PE=2 SV=2//5.88892e-16
95	Ciclev10011092m.v1.0	33.30	0.00	6.02	7.45E-15	4.57E-14	sp|Q9M353|CHX20_ARATH Cation/H(+) antiporter 20 OS=Arabidopsis thaliana GN=CHX20 PE=2 SV=1//0
96	Ciclev10022899m.v1.0	33.23	0.00	6.01	7.92E-15	4.85E-14	Not found
97	Ciclev10019121m.v1.0	16.43	0.00	6.00	4.61E-08	1.36E-07	Not found
98	Ciclev10030602m.v1.0	32.85	0.00	6.00	1.08E-14	6.50E-14	sp|C0LGP4|Y3475_ARATH Probable LRR receptor-like serine/threonine-protein kinase At3g47570 OS=Arabidopsis thaliana GN=At3g47570 PE=1 SV=1//2.74027e-11
99	Ciclev10032743m.v1.0	81.98	1.29	5.99	2.68E-34	5.17E-33	sp|O80852|GSTF9_ARATH Glutathione S-transferase F9 OS=Arabidopsis thaliana GN=GSTF9 PE=1 SV=1//1.90612e-104
100	Ciclev10020289m.v1.0	31.81	0.00	5.99	2.81E-14	1.63E-13	sp|Q9ZU96|Y2168_ARATH Ankyrin repeat-containing protein At2g01680 OS=Arabidopsis thaliana GN=At2g01680 PE=1 SV=1//6.69508e-12

Table S3. The up-regulated DESs in CLas-infected leaf midribs (compared with CLas-infected fruit pith) involved in biosynthesis of secondary metabolites.

Gene_id	Read count (leaf midribs) data	Read count (fruit pith)	Log2 Fold change.	pvalue	qvalue	Blast swiss prot
Ciclev10004731m.v1.0	134.25	0.00	8.0688	2.048E-44	5.513E-43	sp|P55229|GLGL1_ARATH Glucose-1-phosphate adenylyltransferase large subunit 1, chloroplastic OS=Arabidopsis thaliana GN=ADG2 PE=1 SV=3//0
Ciclev10021095m.v1.0	90.72	0.00	7.4609	6.744E-33	1.202E-31	sp|Q94LX1|CLH1_CITUN Chlorophyllase-1, chloroplastic OS=Citrus unshiu PE=2 SV=1//2.91505e-117
Ciclev10006614m.v1.0	41.89	0.00	7.346	2.997E-16	2.077E-15	sp|Q43077|AMO_PEA Primary amine oxidase OS=Pisum sativum PE=1 SV=1//9.54426e-27
Ciclev10031329m.v1.0	127.31	1.03	6.9497	4.486E-48	1.336E-46	sp|Q9XF43|KCS6_ARATH 3-ketoacyl-CoA synthase 6 OS=Arabidopsis thaliana GN=CUT1 PE=1 SV=1//0
Ciclev10028831m.v1.0	156.72	1.54	6.6647	1.898E-60	8.171E-59	sp|P06215|CHIT_PHAVU Endochitinase OS=Phaseolus vulgaris PE=1 SV=1//4.09785e-111
Ciclev10022063m.v1.0	101.47	1.03	6.6225	6.419E-40	1.544E-38	sp|O04138|CHI4_ORYSJ Chitinase 4 OS=Oryza sativa subsp. japonica GN=Cht4 PE=2 SV=2//3.40713e-91
Ciclev10028195m.v1.0	1987.98	27.29	6.1867	0	0	sp|Q00081|GLGL1_SOLTU Glucose-1-phosphate adenylyltransferase large subunit 1 (Fragment) OS=Solanum tuberosum GN=AGPS1 PE=2 SV=1//0
Ciclev10033930m.v1.0	35.84	0.00	6.121	9.405E-16	6.242E-15	sp|Q8W3Z1|BAMS_BETPL Beta-amyrin synthase OS=Betula platyphylla GN=OSCBPY PE=1 SV=1//0
Ciclev10002362m.v1.0	70.78	1.03	6.1029	1.323E-29	2.039E-28	sp|P42820|CHIP_BETVU Acidic endochitinase SP2 OS=Beta vulgaris GN=SP2 PE=1 SV=1//5.18762e-72

Ciclev10012221m.v1.0	29.12	0.00	5.8639	2.629E-13	1.402E-12	sp|P50165|TRNH_DATST Tropinone reductase homolog OS=Datura stramonium PE=2 SV=1//2.56728e-123
Ciclev10025382m.v1.0	28.07	0.00	5.7687	5.887E-13	3.029E-12	sp|Q42600|C84A1_ARATH Cytochrome P450 84A1 OS=Arabidopsis thaliana GN=CYP84A1 PE=2 SV=1//0
Ciclev10000848m.v1.0	189.35	3.60	5.7151	2.347E-78	1.439E-76	sp|O22203|C98A3_ARATH Cytochrome P450 98A3 OS=Arabidopsis thaliana GN=CYP98A3 PE=1 SV=1//0
Ciclev10028959m.v1.0	293.88	5.66	5.6972	1.1E-120	1.18E-118	sp|P06215|CHIT_PHAVU Endochitinase OS=Phaseolus vulgaris PE=1 SV=1//6.905e-104
Ciclev10020692m.v1.0	396.85	7.98	5.6358	6.97E-163	1.13E-160	sp|Q9LRR9|GLO1_ARATH Peroxisomal (S)-2-hydroxy-acid oxidase GLO1 OS=Arabidopsis thaliana GN=GLO1 PE=1 SV=1//0
Ciclev10025492m.v1.0	10.60	0.00	5.3639	7.5E-06	1.607E-05	sp|Q70E96|AL3F1_ARATH Aldehyde dehydrogenase family 3 member F1 OS=Arabidopsis thaliana GN=ALDH3F1 PE=2 SV=2//0
Ciclev10025807m.v1.0	20.53	0.00	5.3599	4.556E-10	1.712E-09	sp|Q9FSC0|ACS2_RUTGR Acridone synthase 2 OS=Ruta graveolens GN=ACS2 PE=1 SV=1//0
Ciclev10030182m.v1.0	17.70	0.00	5.1453	6.075E-09	2.005E-08	sp|Q9LW83|CE101_ARATH G-type lectin S-receptor-like serine/threonine-protein kinase CES101 OS=Arabidopsis thaliana GN=CES101 PE=2 SV=2//1.46348e-88
Ciclev10010729m.v1.0	18.07	0.00	5.133	4.175E-09	1.41E-08	sp|Q95M17|CHIA_BOVIN Acidic mammalian chitinase OS=Bos taurus GN=CHIA PE=1 SV=1//3.59489e-45
Ciclev10029456m.v1.0	134.85	3.86	5.1258	5.056E-58	2.061E-56	sp|Q56Y11|DDPS2_ARATH Dehydrodolichyl diphosphate synthase 2 OS=Arabidopsis thaliana GN=At5g58770 PE=2 SV=2//1.08278e-57
Ciclev10030771m.v1.0	67.05	2.06	5.0247	7.973E-30	1.24E-28	sp|Q8W3Z1|BAMS_BETPL Beta-amyrin synthase OS=Betula platyphylla GN=OSCBPY PE=1 SV=1//0
Ciclev10022123m.v1.0	33.23	1.03	5.0118	1.382E-15	9.014E-15	sp|P29023|CHIB_MAIZE Endochitinase B (Fragment) OS=Zea mays PE=1 SV=1//1.01598e-75

Ciclev10015207m.v1.0	48.01	1.54	4.9579	7.312E-22	7.406E-21	sp|O50046|TRPB_CAMAC Tryptophan synthase beta chain 2, chloroplastic OS=Camptotheca acuminata GN=TSB PE=2 SV=1//0
Ciclev10011989m.v1.0	15.38	0.00	4.9431	5.324E-08	1.56E-07	sp|Q66HG4|GALM_RAT Aldose 1-epimerase OS=Rattus norvegicus GN=Galm PE=1 SV=1//3.90961e-78
Ciclev10028814m.v1.0	7.32	0.00	4.8289	0.0001713	0.0002872	sp|P19446|MDHG_CITLA Malate dehydrogenase, glyoxysomal OS=Citrullus lanatus PE=1 SV=1//0
Ciclev10007953m.v1.0	35.39	1.29	4.781	1.348E-16	9.637E-16	sp|Q570B4|KCS10_ARATH 3-ketoacyl-CoA synthase 10 OS=Arabidopsis thaliana GN=FDH PE=1 SV=2//0
Ciclev10028964m.v1.0	104.83	3.86	4.7626	5.577E-46	1.566E-44	sp|P85084|CHIT_CARPA Endochitinase OS=Carica papaya PE=1 SV=1//1.31916e-102
Ciclev10033766m.v1.0	12.69	0.00	4.666	7.142E-07	1.792E-06	sp|A8C980|GERS_RHISY Germanicol synthase OS=Rhizophora stylosa GN=M1 PE=1 SV=1//0
Ciclev10028774m.v1.0	12.84	0.00	4.6404	6.117E-07	1.55E-06	sp|Q9M439|BCAT2_ARATH Branched-chain-amino-acid aminotransferase 2, chloroplastic OS=Arabidopsis thaliana GN=BCAT2 PE=1 SV=1//1.04448e-158
Ciclev10032697m.v1.0	18.37	0.00	4.5717	2.415E-09	8.398E-09	sp|Q9ZWR1|CFI_CITSI Chalcone--flavonone isomerase OS=Citrus sinensis GN=CHI PE=2 SV=1//2.22362e-155
Ciclev10015924m.v1.0	60.40	2.57	4.5522	2.706E-27	3.701E-26	sp|Q9LSY7|PER30_ARATH Peroxidase 30 OS=Arabidopsis thaliana GN=PER30 PE=1 SV=1//1.27016e-152
Ciclev10001399m.v1.0	71.08	3.09	4.524	7.858E-32	1.336E-30	sp|Q9SK87|ADHL2_ARATH Alcohol dehydrogenase-like 2 OS=Arabidopsis thaliana GN=At1g22440 PE=2 SV=1//6.65423e-176
Ciclev10032730m.v1.0	29.42	1.29	4.5143	4.238E-14	2.444E-13	sp|B7FA90|HPT1_ORYSJ Probable homogentisate phytyltransferase 1, chloroplastic OS=Oryza sativa subsp. japonica GN=HPT1 PE=2 SV=1//4.92606e-85
Ciclev10023297m.v1.0	11.27	0.00	4.495	2.92E-06	6.724E-06	sp|O04138|CHI4_ORYSJ Chitinase 4 OS=Oryza sativa subsp. japonica GN=Cht4 PE=2 SV=2//5.93323e-89

Ciclev10006605m.v1.0	23.22	1.03	4.4949	1.93E-11	8.478E-11	sp|A3C4S4|GME1_ORYSJ GDP-mannose 3,5-epimerase 1 OS=Oryza sativa subsp. japonica GN=GME-1 PE=1 SV=1//0
Ciclev10001618m.v1.0	46.07	2.06	4.4833	3.285E-21	3.185E-20	sp|Q7XWU3|CADH6_ORYSJ Probable cinnamyl alcohol dehydrogenase 6 OS=Oryza sativa subsp. japonica GN=CAD6 PE=2 SV=2//0
Ciclev10021010m.v1.0	15.68	0.00	4.3434	3.482E-08	1.043E-07	sp|A7QEU4|PER5_VITVI Peroxidase 5 OS=Vitis vinifera GN=GSVIVT00037159001 PE=1 SV=2//2.57516e-104
Ciclev10018100m.v1.0	8.74	0.00	4.127	3.92E-05	7.384E-05	sp|P54769|TYDC2_PAPSO Tyrosine/DOPA decarboxylase 2 OS=Papaver somniferum GN=TYDC2 PE=2 SV=1//0
Ciclev10001726m.v1.0	22.33	1.29	4.1162	4.929E-11	2.081E-10	sp|Q96520|PER12_ARATH Peroxidase 12 OS=Arabidopsis thaliana GN=PER12 PE=1 SV=1//6.12414e-150
Ciclev10031967m.v1.0	8.06	0.00	4.0115	7.948E-05	0.0001419	sp|Q8W3Z1|BAMS_BETPL Beta-amyrin synthase OS=Betula platyphylla GN=OSCBPY PE=1 SV=1//4.58637e-178
Ciclev10016309m.v1.0	8.14	0.00	3.9823	7.411E-05	0.0001331	sp|Q9ZU38|RPIA_ARATH Probable ribose-5-phosphate isomerase OS=Arabidopsis thaliana GN=At2g01290 PE=2 SV=1//4.85558e-115
Ciclev10032478m.v1.0	11.95	0.00	3.9511	1.595E-06	3.822E-06	sp|B5BSX1|BAMO_GLYUR Beta-amyrin 11-oxidase OS=Glycyrrhiza uralensis GN=CYP88D6 PE=1 SV=1//4.11313e-97
Ciclev10004484m.v1.0	7.84	0.00	3.9284	0.0001018	0.0001785	sp|Q43077|AMO_PEA Primary amine oxidase OS=Pisum sativum PE=1 SV=1//0
Ciclev10011855m.v1.0	54.51	3.86	3.819	1.492E-24	1.79E-23	sp|P46275|F16P1_PEA Fructose-1,6-bisphosphatase, chloroplastic OS=Pisum sativum GN=FBP PE=1 SV=2//0
Ciclev10020342m.v1.0	7.24	0.00	3.8141	0.0001932	0.000321	sp|Q9SQT8|DHQSD_ARATH Bifunctional 3-dehydroquinate dehydratase/shikimate dehydrogenase, chloroplastic OS=Arabidopsis thaliana GN=EMB3004 PE=1 SV=1//5.39541e-127
Ciclev10004154m.v1.0	459.27	33.21	3.7895	1.75E-193	3.58E-191	sp|Q9FNB0|CHLH_ARATH Magnesium-chelatase subunit ChlH, chloroplastic OS=Arabidopsis thaliana GN=CHLH PE=1 SV=1//0
Ciclev10024601m.v1.0	14.19	1.03	3.784	1.847E-07	5.039E-07	sp|Q9SYK0|HEXO2_ARATH Beta-hexosaminidase 2 OS=Arabidopsis thaliana GN=HEXO2 PE=1 SV=1//3.72587e-08

Ciclev10026670m.v1.0	6.65	0.00	3.7323	0.0003644	0.0005717	sp|Q8GSM7|HST_TOBAC Shikimate O-hydroxycinnamoyltransferase OS=Nicotiana tabacum GN=HST PE=1 SV=1//8.65251e-45
Ciclev10031003m.v1.0	64.14	4.89	3.7127	1.768E-28	2.582E-27	sp|O49675|CCD4_ARATH Probable carotenoid cleavage dioxygenase 4, chloroplastic OS=Arabidopsis thaliana GN=CCD4 PE=1 SV=1//0
Ciclev10012493m.v1.0	6.65	0.00	3.6899	0.0003694	0.0005786	sp|P50165|TRNH_DATST Tropinone reductase homolog OS=Datura stramonium PE=2 SV=1//8.99001e-105
Ciclev10023951m.v1.0	9.33	0.00	3.595	2.557E-05	4.974E-05	sp|Q9M2E2|SDR1_ARATH (+)-neomenthol dehydrogenase OS=Arabidopsis thaliana GN=SDR1 PE=1 SV=1//1.0768e-69
Ciclev10031200m.v1.0	27.93	2.32	3.5911	3.314E-13	1.745E-12	sp|Q8L4B0|GAUTF_ARATH Probable galacturonosyltransferase 15 OS=Arabidopsis thaliana GN=GAUT15 PE=2 SV=1//0
Ciclev10011842m.v1.0	355.48	31.15	3.5123	4.18E-148	5.95E-146	sp|Q6SJV8|CRD1_GOSHI Magnesium-protoporphyrin IX monomethyl ester [oxidative] cyclase, chloroplastic OS=Gossypium hirsutum GN=CRD1 PE=2 SV=2//0
Ciclev10024236m.v1.0	8.74	0.00	3.4995	4.884E-05	9.027E-05	sp|Q9M9V6|ICS2_ARATH Isochorismate synthase 2, chloroplastic OS=Arabidopsis thaliana GN=ICS2 PE=2 SV=2//0
Ciclev10011497m.v1.0	5.60	0.00	3.4854	0.001154	0.0016305	sp|Q9FF18|C7351_ARATH Cytokinin hydroxylase OS=Arabidopsis thaliana GN=CYP735A1 PE=1 SV=1//0
Ciclev10019612m.v1.0	30.91	2.83	3.4482	2.416E-14	1.416E-13	sp|Q9FNF2|SSY1_ARATH Starch synthase 1, chloroplastic/amyloplastic OS=Arabidopsis thaliana GN=SS1 PE=2 SV=1//0
Ciclev10015779m.v1.0	5.38	0.00	3.3841	0.0015081	0.0020651	sp|Q9LEH3|PER15_IPOBA Peroxidase 15 OS=Ipomoea batatas GN=pod PE=1 SV=1//5.78559e-142
Ciclev10019997m.v1.0	18.14	1.80	3.3316	5.966E-09	1.972E-08	sp|Q42954|KPYC_TOBAC Pyruvate kinase, cytosolic isozyme OS=Nicotiana tabacum PE=2 SV=1//0
Ciclev10011063m.v1.0	370.27	37.07	3.3201	4.15E-152	6.12E-150	sp|Q9LZS3|GLGB2_ARATH 1,4-alpha-glucan-branching enzyme 2-2, chloroplastic/amyloplastic OS=Arabidopsis thaliana GN=SBE2.2 PE=1 SV=1//0

Ciclev10015485m.v1.0	5.08	0.00	3.3016	0.0021133	0.0028015	sp|Q56YA5|SGAT_ARATH Serine--glyoxylate aminotransferase OS=Arabidopsis thaliana GN=AGT1 PE=1 SV=2//0
Ciclev10005414m.v1.0	14.86	1.54	3.2658	1.516E-07	4.179E-07	sp|Q94ID2|IPT5_ARATH Adenylate isopentenyltransferase 5, chloroplastic OS=Arabidopsis thaliana GN=IPT5 PE=1 SV=2//1.30222e-133
Ciclev10015692m.v1.0	4.63	0.00	3.1683	0.0035232	0.0044457	sp|Q9ZPS3|DCE4_ARATH Glutamate decarboxylase 4 OS=Arabidopsis thaliana GN=GAD4 PE=2 SV=1//0
Ciclev10002102m.v1.0	4.55	0.00	3.1449	0.0038389	0.0047952	sp|Q9FH04|ADHL7_ARATH Alcohol dehydrogenase-like 7 OS=Arabidopsis thaliana GN=At5g42250 PE=2 SV=1//5.9218e-63
Ciclev10004891m.v1.0	240.50	27.29	3.1395	6.05E-98	5.029E-96	sp|Q42961|PGKH_TOBAC Phosphoglycerate kinase, chloroplastic OS=Nicotiana tabacum PE=2 SV=1//0
Ciclev10019925m.v1.0	53.91	6.18	3.125	2.876E-23	3.203E-22	sp|Q9SM43|VDE_SPIOL Violaxanthin de-epoxidase, chloroplastic OS=Spinacia oleracea GN=VDE1 PE=1 SV=2//0
Ciclev10020814m.v1.0	110.36	14.68	2.9107	7.503E-45	2.045E-43	sp|P46484|COMT1_EUCGU Caffeic acid 3-O-methyltransferase OS=Eucalyptus gunnii GN=OMT PE=2 SV=1//0
Ciclev10008669m.v1.0	14.49	2.06	2.8141	4.194E-07	1.088E-06	sp|Q13231|CHIT1_HUMAN Chitotriosidase-1 OS=Homo sapiens GN=CHIT1 PE=1 SV=1//4.27268e-47
Ciclev10028326m.v1.0	14.11	2.06	2.7764	6.301E-07	1.592E-06	sp|Q43117|KPYA_RICCO Pyruvate kinase isozyme A, chloroplastic OS=Ricinus communis PE=1 SV=1//0
Ciclev10026072m.v1.0	64.96	9.53	2.7696	1.216E-26	1.618E-25	sp|Q9SZB9|PER47_ARATH Peroxidase 47 OS=Arabidopsis thaliana GN=PER47 PE=2 SV=2//1.24796e-149
Ciclev10004473m.v1.0	14.56	2.32	2.6516	5.299E-07	1.354E-06	sp|Q43077|AMO_PEA Primary amine oxidase OS=Pisum sativum PE=1 SV=1//0
Ciclev10008520m.v1.0	326.74	52.78	2.6301	2.21E-124	2.58E-122	sp|Q41249|PORA_CUCSA Protochlorophyllide reductase, chloroplastic OS=Cucumis sativus GN=PORA PE=2 SV=1//0
Ciclev10004736m.v1.0	9.56	1.54	2.6292	4.985E-05	9.201E-05	sp|O48780|KCS11_ARATH 3-ketoacyl-CoA synthase 11 OS=Arabidopsis thaliana GN=KCS11 PE=1 SV=1//0

Ciclev10015856m.v1.0	6.27	1.03	2.6065	0.0010372	0.0014824	sp|Q9ZWQ9|FLS_CITUN Flavonol synthase/flavanone 3-hydroxylase OS=Citrus unshiu GN=FLS PE=1 SV=1//7.94955e-172
Ciclev10014887m.v1.0	13.22	2.32	2.5118	2.3E-06	5.384E-06	sp|O80690|BGL46_ARATH Beta-glucosidase 46 OS=Arabidopsis thaliana GN=BGLU46 PE=1 SV=2//0
Ciclev10031201m.v1.0	12.99	2.32	2.4872	2.939E-06	6.764E-06	sp|O48676|U74B1_ARATH UDP-glycosyltransferase 74B1 OS=Arabidopsis thaliana GN=UGT74B1 PE=1 SV=1//1.76023e-162
Ciclev10019920m.v1.0	14.34	2.57	2.4772	9.262E-07	2.283E-06	sp|P31531|1A1C_SOYBN 1-aminocyclopropane-1-carboxylate synthase OS=Glycine max GN=ACS1 PE=2 SV=1//0
Ciclev10001943m.v1.0	55.78	10.04	2.4737	3.834E-22	3.979E-21	sp|Q96DG6|CMBL_HUMAN Carboxymethylenebutenolidase homolog OS=Homo sapiens GN=CMBL PE=1 SV=1//4.08809e-25
Ciclev10026028m.v1.0	130.96	24.20	2.436	2.059E-49	6.624E-48	sp|Q9ZWQ9|FLS_CITUN Flavonol synthase/flavanone 3-hydroxylase OS=Citrus unshiu GN=FLS PE=1 SV=1//0
Ciclev10011175m.v1.0	38.00	7.21	2.3983	2.139E-15	1.368E-14	sp|P45730|PALY_POPTR Phenylalanine ammonia-lyase OS=Populus trichocarpa GN=PAL PE=2 SV=1//0
Ciclev10026121m.v1.0	9.41	1.80	2.3841	8.089E-05	0.0001443	sp|Q40784|AAPC_CENCI Putative glucose-6-phosphate 1-epimerase OS=Cenchrus ciliaris PE=2 SV=1//2.39487e-41
Ciclev10007733m.v1.0	177.33	36.30	2.2883	1.724E-64	7.952E-63	sp|O24145|4CL1_TOBAC 4-coumarate--CoA ligase 1 OS=Nicotiana tabacum GN=4CL1 PE=2 SV=1//0
Ciclev10016176m.v1.0	23.15	4.89	2.2423	1.079E-09	3.908E-09	sp|P48261|TRPG_CYAPA Anthranilate synthase component II OS=Cyanophora paradoxa GN=trpG PE=3 SV=1//1.37682e-70
Ciclev10015582m.v1.0	7.24	1.54	2.2291	0.0006593	0.0009812	sp|P08196|PSY1_SOLLC Phytoene synthase 1, chloroplastic OS=Solanum lycopersicum GN=PSY1 PE=1 SV=2//6.10667e-135
Ciclev10021969m.v1.0	35.99	7.72	2.2201	3.317E-14	1.92E-13	sp|Q8LAH8|NDK4_ARATH Nucleoside diphosphate kinase IV, chloroplastic/mitochondrial OS=Arabidopsis thaliana GN=NDK4 PE=1 SV=2//4.02118e-128

Ciclev10022050m.v1.0	18.97	4.12	2.2028	3.879E-08	1.155E-07	sp|O04138|CHI4_ORYSJ Chitinase 4 OS=Oryza sativa subsp. japonica GN=Cht4 PE=2 SV=2//1.65072e-95
Ciclev10026918m.v1.0	22.92	5.15	2.1543	1.846E-09	6.503E-09	sp|O65922|CAMT2_POPTR Caffeoyl-CoA O-methyltransferase 2 OS=Populus trichocarpa GN=CCOAOMT2 PE=2 SV=1//4.41196e-16
Ciclev10008483m.v1.0	6.72	1.54	2.121	0.0011852	0.0016692	sp|Q8VWJ1|HPT1_ARATH Homogentisate phytyltransferase 1, chloroplastic OS=Arabidopsis thaliana GN=VTE2-1 PE=2 SV=1//3.45423e-85
Ciclev10019770m.v1.0	13.44	3.09	2.121	4.53E-06	1.008E-05	sp|P51094|UFOG_VITVI Anthocyanidin 3-O-glucosyltransferase 2 OS=Vitis vinifera GN=UFGT PE=1 SV=2//4.84349e-178
Ciclev10000835m.v1.0	47.94	11.07	2.1143	4.998E-18	3.974E-17	sp|P27608|AROF_TOBAC Phospho-2-dehydro-3-deoxyheptonate aldolase 1, chloroplastic OS=Nicotiana tabacum GN=DHAPS-1 PE=2 SV=1//0
Ciclev10008012m.v1.0	5.45	1.29	2.082	0.0036476	0.0045863	sp|Q9FIU7|BGL41_ARATH Putative beta-glucosidase 41 OS=Arabidopsis thaliana GN=BGLU41 PE=3 SV=2//1.14886e-39
Ciclev10010975m.v1.0	181.74	44.03	2.0454	1.02E-62	4.58E-61	sp|P04045|PHSL1_SOLTU Alpha-1,4 glucan phosphorylase L-1 isozyme, chloroplastic/amyloplastic OS=Solanum tuberosum PE=1 SV=2//0
Ciclev10028147m.v1.0	220.26	53.81	2.0333	2.082E-75	1.222E-73	sp|Q9FYC2|PAO_ARATH Pheophorbide a oxygenase, chloroplastic OS=Arabidopsis thaliana GN=PAO PE=1 SV=1//0
Ciclev10028226m.v1.0	9.48	2.32	2.0329	0.0001377	0.0002358	sp|A8G9J4|GPMB_SERP5 Probable phosphoglycerate mutase GpmB OS=Serratia proteamaculans (strain 568) GN=gpmB PE=3 SV=1//5.06265e-17
Ciclev10024238m.v1.0	7.32	1.80	2.0215	0.0008253	0.0012033	sp|Q39108|GGR_ARATH Heterodimeric geranylgeranyl pyrophosphate synthase small subunit, chloroplastic OS=Arabidopsis thaliana GN=GGR PE=1 SV=2//1.06847e-09
Ciclev10033793m.v1.0	16.65	4.12	2.015	4.64E-07	1.197E-06	sp|B5BSX1|BAMO_GLYUR Beta-amyrin 11-oxidase OS=Glycyrrhiza uralensis GN=CYP88D6 PE=1 SV=1//1.01504e-49
Ciclev10020061m.v1.0	86.99	22.40	1.9573	2.678E-30	4.307E-29	sp|Q9CA67|CHLP_ARATH Geranylgeranyl diphosphate reductase, chloroplastic OS=Arabidopsis thaliana GN=CHLP PE=1 SV=1//0

Ciclev10031862m.v1.0	39.72	10.30	1.9475	1.163E-14	6.993E-14	sp|B5BSX1|BAMO_GLYUR Beta-amyrin 11-oxidase OS=Glycyrrhiza uralensis GN=CYP88D6 PE=1 SV=1//1.20298e-102
Ciclev10018950m.v1.0	42.63	11.07	1.9452	1.288E-15	8.425E-15	sp|Q07123|AMO2_ARTS1 Copper methylamine oxidase OS=Arthrobacter sp. (strain P1) GN=maoII PE=1 SV=1//1.03603e-168
Ciclev10000842m.v1.0	69.44	18.28	1.9255	2.479E-24	2.927E-23	sp|P55233|GLGL1_BETVU Glucose-1-phosphate adenylyltransferase large subunit, chloroplastic/amyloplastic OS=Beta vulgaris GN=AGPS1 PE=2 SV=1//0
Ciclev10031855m.v1.0	26.28	6.95	1.9187	3.943E-10	1.496E-09	sp|Q9FH04|ADHL7_ARATH Alcohol dehydrogenase-like 7 OS=Arabidopsis thaliana GN=At5g42250 PE=2 SV=1//4.09278e-85
Ciclev10025755m.v1.0	352.20	93.97	1.906	1.01E-115	1.04E-113	sp|Q9ZRF1|MTDH_FRAAN Probable mannitol dehydrogenase OS=Fragaria ananassa GN=CAD PE=2 SV=1//2.8565e-160
Ciclev10010416m.v1.0	13.37	3.60	1.8906	8.795E-06	1.865E-05	sp|P38605|CAS1_ARATH Cycloartenol synthase OS=Arabidopsis thaliana GN=CAS1 PE=1 SV=2//0
Ciclev10030821m.v1.0	63.62	17.25	1.8828	3.416E-22	3.554E-21	sp|Q42667|PALY_CITLI Phenylalanine ammonia-lyase OS=Citrus limon GN=PAL6 PE=2 SV=1//0
Ciclev10021564m.v1.0	80.04	22.14	1.854	2.652E-27	3.632E-26	sp|Q43157|RPE_SPIOL Ribulose-phosphate 3-epimerase, chloroplastic OS=Spinacia oleracea GN=RPE PE=1 SV=1//5.3858e-172
Ciclev10028359m.v1.0	18.59	5.15	1.8522	1.836E-07	5.011E-07	sp|Q9SGD6|AROD6_ARATH Arogenate dehydratase/prephenate dehydratase 6, chloroplastic OS=Arabidopsis thaliana GN=ADT6 PE=1 SV=1//0
Ciclev10031341m.v1.0	19.64	5.66	1.7936	1.06E-07	2.988E-07	sp|B5BSX1|BAMO_GLYUR Beta-amyrin 11-oxidase OS=Glycyrrhiza uralensis GN=CYP88D6 PE=1 SV=1//2.96388e-180
Ciclev10028384m.v1.0	662.44	194.13	1.7708	4.82E-208	1.04E-205	sp|Q94B35|ISPH_ARATH 4-hydroxy-3-methylbut-2-enyl diphosphate reductase, chloroplastic OS=Arabidopsis thaliana GN=ISPH PE=2 SV=1//0
Ciclev10025280m.v1.0	30.16	9.01	1.7431	6.129E-11	2.559E-10	sp|O24145|4CL1_TOBAC 4-coumarate--CoA ligase 1 OS=Nicotiana tabacum GN=4CL1 PE=2 SV=1//0
Ciclev10026035m.v1.0	17.10	5.15	1.7314	8.845E-07	2.188E-06	sp|Q42580|PER21_ARATH Peroxidase 21 OS=Arabidopsis thaliana GN=PER21 PE=1 SV=1//2.11891e-174

Ciclev10000592m.v1.0	244.46	73.63	1.7311	4.07E-77	2.458E-75	sp|Q0GZS3|USP_CUCME UDP-sugar pyrophospharylase OS=Cucumis melo GN=USP PE=1 SV=1//0
Ciclev10005157m.v1.0	6.50	2.06	1.6571	0.0027389	0.0035386	sp|Q39110|GAOX1_ARATH Gibberellin 20 oxidase 1 OS=Arabidopsis thaliana GN=20ox1 PE=2 SV=2//0
Ciclev10007114m.v1.0	36.66	11.84	1.6302	1.378E-12	6.764E-12	sp|P28554|CRTI_SOLLC Phytoene dehydrogenase, chloroplastic/chromoplastic OS=Solanum lycopersicum GN=PDS PE=2 SV=1//6.06527e-161
Ciclev10029051m.v1.0	12.69	4.12	1.6235	3.111E-05	5.958E-05	sp|Q9ASX2|TRNH1_ARATH Tropinone reductase homolog At1g07440 OS=Arabidopsis thaliana GN=At1g07440 PE=1 SV=1//3.76843e-129
Ciclev10026185m.v1.0	82.95	27.29	1.6039	2.556E-26	3.353E-25	sp|Q9ZRF1|MTDH_FRAAN Probable mannitol dehydrogenase OS=Fragaria ananassa GN=CAD PE=2 SV=1//7.88698e-143
Ciclev10002980m.v1.0	13.29	4.38	1.6024	2.162E-05	4.266E-05	sp|P42802|INO1_CITPA Inositol-3-phosphate synthase OS=Citrus paradisi PE=3 SV=1//1.41406e-10
Ciclev10023611m.v1.0	38.15	12.62	1.5966	6.481E-13	3.318E-12	sp|P27054|CHI4_PHAVU Endochitinase PR4 OS=Phaseolus vulgaris GN=CHI4 PE=2 SV=1//6.34778e-69
Ciclev10028076m.v1.0	59.28	20.08	1.5617	5.066E-19	4.241E-18	sp|Q9STG9|ASE2_ARATH Amidophosphoribosyltransferase 2, chloroplastic OS=Arabidopsis thaliana GN=ASE2 PE=1 SV=1//0
Ciclev10027684m.v1.0	60.11	20.60	1.5451	3.649E-19	3.098E-18	sp|Q7G193|ALDO1_ARATH Indole-3-acetaldehyde oxidase OS=Arabidopsis thaliana GN=AAO1 PE=1 SV=2//0
Ciclev10026731m.v1.0	32.26	11.07	1.5428	5.689E-11	2.387E-10	sp|Q43095|CAMT_POPTM Caffeoyl-CoA O-methyltransferase OS=Populus tremuloides PE=2 SV=1//1.04177e-96
Ciclev10002135m.v1.0	70.34	24.20	1.5391	4.091E-22	4.235E-21	sp|Q9ZU38|RPIA_ARATH Probable ribose-5-phosphate isomerase OS=Arabidopsis thaliana GN=At2g01290 PE=2 SV=1//2.69515e-99
Ciclev10018991m.v1.0	498.32	171.47	1.5391	4.27E-146	5.98E-144	sp|Q43848|TKTC_SOLTU Transketolase, chloroplastic OS=Solanum tuberosum PE=2 SV=1//0
Ciclev10017814m.v1.0	18.59	6.44	1.5303	6.925E-07	1.741E-06	sp|Q8VYJ1|MENE_ARATH 2-succinylbenzoate--CoA ligase, chloroplastic/peroxisomal OS=Arabidopsis thaliana GN=AAE14 PE=1 SV=1//0

Ciclev10028614m.v1.0	21.13	7.47	1.5008	1.406E-07	3.895E-07	sp|A7QJG1|METK3_VITVI S-adenosylmethionine synthase 3 OS=Vitis vinifera GN=METK3 PE=3 SV=1//0
Ciclev10008332m.v1.0	133.80	47.37	1.4979	5.067E-40	1.23E-38	sp|Q8VYG2|GALAK_ARATH Galacturonokinase OS=Arabidopsis thaliana GN=GALAK PE=1 SV=1//0
Ciclev10008410m.v1.0	29.72	10.56	1.4932	4.521E-10	1.701E-09	sp|Q38932|LCYE_ARATH Lycopene epsilon cyclase, chloroplastic OS=Arabidopsis thaliana GN=LUT2 PE=1 SV=2//1.07953e-169
Ciclev10000921m.v1.0	532.89	193.10	1.4645	4E-152	6E-150	sp|Q43054|TCMO_POPKI Trans-cinnamate 4-monooxygenase OS=Populus kitakamiensis GN=CYP73A16 PE=2 SV=1//0
Ciclev10031373m.v1.0	52.79	19.31	1.4509	1.619E-16	1.147E-15	sp|Q9FWA3|6GPD3_ARATH 6-phosphogluconate dehydrogenase, decarboxylating 3 OS=Arabidopsis thaliana GN=At3g02360 PE=2 SV=1//0
Ciclev10031858m.v1.0	39.72	14.68	1.4365	9.642E-13	4.833E-12	sp|Q43316|HEM3_ARATH Porphobilinogen deaminase, chloroplastic OS=Arabidopsis thaliana GN=HEMC PE=1 SV=1//0
Ciclev10017811m.v1.0	17.02	6.44	1.4032	3.442E-06	7.839E-06	sp|Q9LW20|SKL1_ARATH Probable inactive shikimate kinase like 1, chloroplastic OS=Arabidopsis thaliana GN=SKL1 PE=2 SV=1//2.80357e-76
Ciclev10025933m.v1.0	9.48	3.60	1.3955	0.0005405	0.000819	sp|Q9ZRF1|MTDH_FRAAN Probable mannitol dehydrogenase OS=Fragaria ananassa GN=CAD PE=2 SV=1//0
Ciclev10018875m.v1.0	14.86	5.66	1.3913	1.508E-05	3.064E-05	sp|O04408|KSA_PEA Ent-copalyl diphosphate synthase, chloroplastic OS=Pisum sativum PE=2 SV=1//0
Ciclev10021162m.v1.0	294.86	115.34	1.3541	1.117E-81	7.327E-80	sp|A2Z3C4|6PGL4_ORYSI Probable 6-phosphogluconolactonase 4, chloroplastic OS=Oryza sativa subsp. indica GN=OsI_031067 PE=3 SV=2//6.64477e-138
Ciclev10015965m.v1.0	113.19	44.54	1.3456	2.415E-32	4.242E-31	sp|P31237|ACCO_ACTDE 1-aminocyclopropane-1-carboxylate oxidase OS=Actinidia deliciosa GN=ACO PE=2 SV=1//0
Ciclev10019836m.v1.0	18.14	7.21	1.3316	2.275E-06	5.331E-06	sp|Q8W250|DXR_ORYSJ 1-deoxy-D-xylulose 5-phosphate reductoisomerase, chloroplastic OS=Oryza sativa subsp. japonica GN=DXR PE=2 SV=2//0
Ciclev10005101m.v1.0	208.02	83.42	1.3183	2.22E-57	8.963E-56	sp|Q42962|PGKY_TOBAC Phosphoglycerate kinase, cytosolic OS=Nicotiana tabacum PE=2 SV=1//0

Ciclev10014741m.v1.0	39.35	15.96	1.3016	4.505E-12	2.107E-11	sp|P52417|GLGS2_VICFA Glucose-1-phosphate adenylyltransferase small subunit 2, chloroplastic OS=Vicia faba GN=AGPP PE=2 SV=1//0
Ciclev10008705m.v1.0	19.94	8.24	1.2748	9.627E-07	2.369E-06	sp|Q9SMZ4|AASS_ARATH Alpha-aminoadipic semialdehyde synthase OS=Arabidopsis thaliana GN=LKR/SDH PE=1 SV=1//0
Ciclev10025338m.v1.0	163.44	68.74	1.2495	2.933E-44	7.867E-43	sp|A2Y3Q5|HEMH_ORYSI Ferrochelatase-2, chloroplastic OS=Oryza sativa subsp. indica GN=HEMH PE=2 SV=2//0
Ciclev10020068m.v1.0	79.44	33.47	1.2471	2.383E-22	2.498E-21	sp|Q56WD9|THIK2_ARATH 3-ketoacyl-CoA thiolase 2, peroxisomal OS=Arabidopsis thaliana GN=PED1 PE=1 SV=2//0
Ciclev10004626m.v1.0	32.85	13.90	1.2406	4.248E-10	1.604E-09	sp|Q9FLW9|PKP2_ARATH Plastidial pyruvate kinase 2 OS=Arabidopsis thaliana GN=PKP2 PE=1 SV=1//0
Ciclev10016114m.v1.0	20.98	9.01	1.2193	6.767E-07	1.703E-06	sp|P17783|MDHM_CITLA Malate dehydrogenase, mitochondrial OS=Citrullus lanatus GN=MMDH PE=1 SV=1//0
Ciclev10020914m.v1.0	22.18	9.53	1.219	3.271E-07	8.638E-07	sp|O80574|DAPB1_ARATH 4-hydroxy-tetrahydrodipicolinate reductase 1, chloroplastic OS=Arabidopsis thaliana GN=DAPB1 PE=2 SV=2//3.36859e-177
Ciclev10028048m.v1.0	127.16	54.84	1.2133	2.589E-34	4.998E-33	sp|Q56078|BGLX_SALTY Periplasmic beta-glucosidase OS=Salmonella typhimurium (strain LT2 / SGSC1412 / ATCC 700720) GN=bglX PE=3 SV=2//1.41326e-71
Ciclev10027291m.v1.0	7.99	3.60	1.1483	0.0025412	0.0033056	sp|Q8VWJ1|HPT1_ARATH Homogentisate phytyltransferase 1, chloroplastic OS=Arabidopsis thaliana GN=VTE2-1 PE=2 SV=1//4.44865e-44
Ciclev10012496m.v1.0	7.39	3.35	1.1431	0.0037319	0.0046755	sp|P50165|TRNH_DATST Tropinone reductase homolog OS=Datura stramonium PE=2 SV=1//5.002e-112
Ciclev10023577m.v1.0	174.20	79.56	1.1307	8.971E-45	2.43E-43	sp|Q9M2E2|SDR1_ARATH (+)-neomenthol dehydrogenase OS=Arabidopsis thaliana GN=SDR1 PE=1 SV=1//5.4789e-60
Ciclev10011124m.v1.0	57.27	26.26	1.1248	9.076E-16	6.038E-15	sp|Q43847|SSY2_SOLTU Granule-bound starch synthase 2, chloroplastic/amyloplastic OS=Solanum tuberosum GN=SS2 PE=1 SV=3//0

Ciclev10001524m.v1.0	68.84	31.93	1.1086	1.62E-18	1.314E-17	sp|A3C4S4|GME1_ORYSJ GDP-mannose 3,5-epimerase 1 OS=Oryza sativa subsp. japonica GN=GME-1 PE=1 SV=1//0
Ciclev10015579m.v1.0	53.09	24.97	1.0879	1.665E-14	9.898E-14	sp|P93841|ISPE_SOLLC 4-diphosphocytidyl-2-C-methyl-D-erythritol kinase, chloroplastic/chromoplastic (Fragment) OS=Solanum lycopersicum GN=ISPE PE=1 SV=1//0
Ciclev10029246m.v1.0	18.97	9.01	1.0735	4.846E-06	1.074E-05	sp|Q2KIP8|HACD2_BOVIN Very-long-chain (3R)-3-hydroxyacyl-[acyl-carrier protein] dehydratase 2 OS=Bos taurus GN=PTPLB PE=2 SV=2//1.75539e-27
Ciclev10014977m.v1.0	136.86	65.65	1.0598	1.884E-34	3.678E-33	sp|Q42954|KPYC_TOBAC Pyruvate kinase, cytosolic isozyme OS=Nicotiana tabacum PE=2 SV=1//0
Ciclev10015153m.v1.0	133.88	64.37	1.0565	1.096E-33	2.044E-32	sp|P37830|G6PD_SOLTU Glucose-6-phosphate 1-dehydrogenase, cytoplasmic isoform OS=Solanum tuberosum GN=G6PDH PE=2 SV=1//0
Ciclev10003601m.v1.0	32.55	15.71	1.0516	2.553E-09	8.847E-09	sp|O64437|INO1_ORYSJ Inositol-3-phosphate synthase OS=Oryza sativa subsp. japonica GN=INO1 PE=2 SV=2//6.86521e-07
Ciclev10025255m.v1.0	24.34	11.84	1.0393	2.796E-07	7.451E-07	sp|Q94AA4|K6PF3_ARATH 6-phosphofructokinase 3 OS=Arabidopsis thaliana GN=PFK3 PE=1 SV=1//0
Ciclev10005632m.v1.0	47.86	23.69	1.0148	8.074E-13	4.082E-12	sp|Q07356|PDS_ARATH 15-cis-phytoene desaturase, chloroplastic/chromoplastic OS=Arabidopsis thaliana GN=PDS PE=1 SV=1//2.13962e-169
